# Enhancing financial data collection and reporting in small businesses through IoT integration: an exploration of IFRS standard

**DOI:** 10.3389/frai.2026.1811519

**Published:** 2026-06-24

**Authors:** Salah Oraby, Amar Johri, Anu Sayal, Kim Mee Chong, Hamad Alhumoudi, Janhvi Jha, Chaithra N

**Affiliations:** 1 Saudi Electronic University, Riyadh, Saudi Arabia; 2 School of Accounting and Finance, Faculty of Business and Law, Taylor’s University, Subang Jaya, Malaysia; 3 Centre for Artificial Intelligence, Madhav Institute of Technology and Science Deemed University, Gwalior, Madhya Pradesh, India; 4 JAIN (Deemed-to-be University), Bengaluru, India

**Keywords:** financial management, IFRS, internet of things (IoT), real-time transaction monitoring, small and medium enterprises (SMEs)

## Abstract

**Introduction:**

The adoption of Internet of Things (IoT) technology offers significant opportunities to improve financial data collection, reporting accuracy, and regulatory compliance in small and medium-sized enterprises (SMEs). Despite its growing use, limited research has examined IoT implementation from the perspective of International Financial Reporting Standards (IFRS) and its implications for financial reporting quality.

**Methods:**

This study employs a hybrid research approach combining a qualitative literature review with secondary dataset-based machine learning analysis using the CIC-IoT-2023 dataset. A structured review of peer-reviewed publications published between 2015 and 2024 was conducted using sources including Google Scholar, Elsevier, IEEE, and Springer. Selected case studies from retail, manufacturing, and agricultural SMEs were also analyzed to identify key factors influencing IoT adoption.

**Results:**

The findings indicate that IoT integration supports real-time transaction monitoring, automated financial reporting, and intelligent inventory management. Evidence from the reviewed literature suggests that IoT adoption is associated with reductions in operating expenses ranging from 35% to 40% and improvements in inventory accuracy of up to 85%. The study further demonstrates that IoT-generated data can enhance the qualitative characteristics of financial reporting defined by IFRS, including comparability, verifiability, timeliness, and understandability. However, several barriers to implementation remain, including cybersecurity risks, high initial investment costs, and inadequate technological infrastructure.

**Discussion:**

This study bridges the gap between IoT adoption research and IFRS-aligned financial reporting practices within resource-constrained SME environments. The proposed framework highlights practical implementation strategies and a phased adoption approach that can assist SMEs in leveraging IoT technologies while maintaining compliance with financial reporting requirements. The findings contribute to both academic research and managerial practice by demonstrating how IoT can support more efficient, transparent, and reliable financial management systems.

## 1 Introduction

The Internet of Things (IoT) has emerged as a key technology paradigm that enables interconnected devices, sensors, and systems to exchange and analyze data with minimal human intervention (Perera et al., 2015). The proper operation of electronics in both real and virtual environments is contingent upon trust (Trend Micro, 2019). By optimizing routine operations, including inventory management, automated invoicing, and operational cost maintenance, the Internet of Things (IoT) can aid small businesses in conserving time and resources (An, 2018; Goodman, 2018).

Financial data collection, as understood from studies undertaken by Musa (2019) signify the four pillars for accuracy in data collection. For small businesses, where the task of maintaining segregated and precise records of financial aspects including but not limited to billings, cashflows, inventory management, balance sheets, resource management and assignment and reporting for predictive, prescriptive and diagnostic analysis, prove to be more labor intensive and error prone due to lack of proper systems. This discrepancy leads to loss of investments and stakeholder trust from financial institutions and large-scale organizations. To assist in these endeavors, IoT supports the digitalization of these mundane tasks that improve the customer experience and business profits for small and medium enterprises (SME; Amaral and Peças, 2021).

As IoT-enabled banking systems become more popular, they also open up new ways to make sure they follow International banking Reporting Standards (IFRS). RFID-enabled inventory monitoring, automated transaction tracking, cloud-based reporting infrastructures, and real-time sensor analytics can all help meet IFRS-aligned requirements for things like valuing inventory, recognizing income, being able to be audited, and making financial data clear. SMEs often have trouble integrating these tools, though, because they do not have the right technical infrastructure, they are worried about security, and they do not have enough resources.

The aim of the present study is therefore to explore the following research question: “How can IoT-enabled technologies support secure, reliable and IFRS compliant financial data management practices in SME environments?” The study also examines the role of cybersecurity-aware IoT frameworks, automated reporting systems, and machine learning-based threat detection models in enhancing financial data reliability and operational efficiency for SMEs.

While the study primarily explores IoT-enabled financial and sustainability frameworks through qualitative literature analysis, it also incorporates a secondary-data experimental component to evaluate cybersecurity risks in IoT ecosystems using the CIC-IoT-2023 dataset. This study examines the ability of Internet of Things (IoT) technology to interact with the financial operations of small enterprises. It specifically investigates the potential applications of contemporary technology frameworks in the accumulation and reporting of financial data. We conduct a comprehensive literature review and examination of current case studies to evaluate the application of the Internet of Things (IoT) in three primary domains: automated reporting systems that utilize XBRL and cloud infrastructure, inventory management that employs RFID and weight-sensor technologies, and real-time transaction monitoring that leverages sensor networks and payment systems. This article assesses the advantages and disadvantages of small enterprises adopting the Internet of Things (IoT), with a particular emphasis on infrastructure requirements, pricing ranges, and security concerns. The proposed hybrid technological framework combines IoT, cloud computing, blockchain, and machine learning to address multiple operational and financial reporting challenges simultaneously. IoT devices enable real-time financial data collection, cloud infrastructure supports scalable storage and accessibility, blockchain improves data integrity and traceability, while machine learning techniques enhance anomaly detection and cybersecurity monitoring. Together, these technologies create an integrated ecosystem capable of improving operational efficiency, financial transparency, and reliability within SME-oriented reporting environments. An examination of effective executions and significant obstacles is the foundation of this methodology. This research offers a thorough examination of the financial operations challenges that are unique to small businesses in the context of the Internet of Things (IoT). It does so by utilizing quantitative evidence of efficiency improvements and expense reductions. The study provides on the current state of research in this area. Additionally, it provides a comprehensive examination of the ways in which small businesses can utilize phased IoT adoption strategies to improve operational efficiency and alleviate resource constraints. This comprehensive review provides understanding of how small enterprises can improve financial data management practices. Develop stakeholder confidence, and enhance operational efficiency while operating under resource constraints by effectively leveraging IoT capabilities.

The remainder of this paper is organized as follows. Section II reviews existing literature relating to IoT adoption, SME financial challenges, and IFRS-oriented reporting frameworks. Section III outlines the research methodology and hybrid analytical approach adopted in the study. Section IV discusses IoT-enabled financial management applications in SMEs, while Section V talks about the benefits of IoT in financial management for small businesses. Section VI examines cybersecurity considerations and machine learning-based threat detection analysis using the CIC-IoT-2023 dataset. Finally, the paper concludes with key findings, limitations, and future research directions.

## 2 Literature review

The literature review focuses on peer-reviewed journal articles, conference papers, industry reports, and scholarly publications published between 2015 and 2024. This timeframe was selected to capture contemporary developments relating to IoT adoption, cloud-based financial systems, blockchain-enabled reporting, cybersecurity, and IFRS-oriented digital financial practices.

Studies were included if they addressed:

- IoT applications in SME financial management
- Automated reporting systems and financial data collection
- IFRS-related reporting technologies
- IoT cybersecurity and operational reliability
- Machine learning applications in IoT environments

Studies unrelated to financial systems, SME operational contexts, or IoT-enabled reporting frameworks were excluded from the review.

### 2.1 IoT in financial services

The banking and financial services sector has created a novel form of customer value as a result of the development of branchless banking services through various communication channels. The proliferation of mobile phones and other wireless devices, such as wearables and sensors, has led to the logical progression of the Internet of Things (IoT) in online banking. This technology has the potential to improve the customer service experience. Investment management, commercial worker compensation, telematics-based insurance, and insurance (both medical and life) are among the industries that could potentially benefit from the Internet of Things. Both the network infrastructure and customer service provided by banks will significantly benefit from the Internet of Things. Effective IoT implementation necessitates the integration of big data analytics and cloud accessibility with IoT structures (Dineshreddy and Gangadharan, 2016; Kassim, 2022).

Blockchain technology is the focal point of financial support for supply chains. This theoretical study focused on blockchain technology and supply chain financing. The systems for management, cash flow, and risk control are examined in the context of the current state of blockchain technology in supply chain finance (Mosallanezhad et al., 2023). The optimization of the supply chain financing risk control system reduces costs, enhances efficiency, and mitigates risks for all parties involved. In order for commercial banks and enterprises to expand, it is necessary to establish a blockchain Internet of Things (IoT) environment that employs advanced processing and shared data (Prajapati et al., 2022). The infrastructure for managing IoT data was demonstrated in this study through the use of blockchain technology and edge computing.

Machine learning and the Internet of Things help financial institutions detect unusual activity, which cybercriminals often target. These advances improve fraud detection and anomaly prevention by collecting data from servers, ATMs, online payment portals, and web applications (Suseendran, 2019). Internet of Things (IoT) streamlines auditing and enables real-time transaction tracking, ensuring accounting transparency. The Internet of Things also helps financial institutions establish audit trails by monitoring all employees’ or business units’ asset and money management and processing responsibilities. This multi-layer IoT implementation shows banking’s revolutionary potential (Bhat et al., 2023; Bisht et al., 2022).

Researchers (Arora and Kaur, 2020) introduced a three-layer smart banking IoT architecture. The physical layer has data collection sensors, while the process layer has cloud, edge computing, and AI-powered analytics. Banking and insurance applications offer feedback, suggestions, and alerts at the service layer. This design shows how IoT can improve banking services.

### 2.2 Small business challenges

Financial issues present a unique set of obstacles for SMEs, as they are the primary concern (Woodward, 2009; Brown and Lee, 2018). In a 1991 study conducted in the United Kingdom by Hall and Young, 86.6% of respondents attributed the failure of their companies to financial issues (Hall and Young, 1991). Financial difficulties remain among the most commonly cited causes of SME failure; Brown and Lee (2018) document persistent financing gaps faced by small enterprises, while Chabalala et al. (2024) observe that the perceived complexity of digital tools and data security concerns further constrain SME willingness to adopt technologies that could alleviate these pressures. Numerous scholars have established substantial correlations between the performance of an enterprise and the results of financial strategy and decision-making (López Salazar et al., 2012). Despite the critical role these aspects play in underpinning organizational strategy, the financial options available to entrepreneurs are substantially restricted by the absence of financial analysis and planning in micro and small enterprises. Strategic financial planning can enable an organization to achieve sustained competitiveness and establish itself as a world-class entity. Financial strategies are designed to enhance and optimize financial management to accomplish the objectives of the company (Mallette, 2006).

Despite the abundance of source data available, there is a substantial lack of actionable data for transformation, purification, integration, modeling, and analysis. Research maintains that this field is noteworthy and warrants further attention. However, there are numerous obstacles to utilizing financial data, including the extensive volume of data, strict real-time requirements, high standards for data quality, security concerns, and reliability issues. Data collection aims to extract significant information by organizing and utilizing large volumes of unstructured data through the use of a variety of collection techniques and methodologies. The financial sector’s data acquisition process is comprised of critical components, including data preparation, accumulation, organization, demand analysis, and allocation (Fourcade and Healy, 2017; Zhang et al., 2023).

The fragmented structure of data systems that contain transaction-specific information presents a substantial challenge for individual financial institutions (Alexander et al., 2017). It is imperative to integrate data from these numerous systems. The aggregation process is impeded by discrepancies in the documentation of financial transactions (Flood et al., 2011). Stein (2013) posits that systemic risk assessments are only effective when data and models are in alignment (Stein, 2013). SMEs receive minimal financial support from commercial banks, which typically provide such funding, according to research. This can be explained by the fact that “traditional banks are not designed to accommodate the type of credit required by small businesses due to their credit assessment methodologies,” and additionally “small businesses possess significant structural deficiencies that render them high-risk borrowers” (Onakoya et al., 2013).

The alignment of IoT capabilities with specific IFRS requirements presents a particularly underexplored opportunity for SMEs. Under IFRS 15 (Revenue from Contracts with Customers), IoT-enabled point-of-sale and real-time transaction monitoring systems can automate the capture of performance obligation completion, supporting accurate and timely revenue recognition (Backrie et al., 2024). IAS 2 (Inventories) requires stock to be measured at the lower of cost and net realizable value; RFID-based inventory systems directly serve this requirement by providing continuous, real-time stock visibility that reduces the risk of overvaluation (Johari and Aziz, 2023; Zhou and Li, 2020). IAS 16 and IAS 36 govern asset measurement and impairment testing, areas where IoT-based predictive maintenance systems can supply the continuous condition data needed to support depreciation calculations (Arena et al., 2022). Finally, IFRS 7 and IFRS 9 require forward-looking risk disclosures and expected credit loss modeling, for which IoT-derived transactional and behavioral data can enrich the inputs available to SME financial managers (Abbasi et al., 2019). Nofel et al. (2024) demonstrate that integrating IoT sensor data with blockchain and XBRL enables real-time, standards-aligned financial reporting, directly addressing the compliance burden faced by small enterprises.

## 3 Hybrid research methodology

The study employs a mixed-method approach. The qualitative part includes a literature synthesis on IoT-enabled financial data collection, reporting issues faced by SMEs and reporting practices aligned to IFRS. The quantitative part utilizes the secondary data from the CIC-IoT-2023 dataset to evaluate the models of the IoT threat detection based on machine learning. This secondary data analysis is included to assess the cybersecurity reliability of IoT systems that are used to support financial data collection and reporting in SMEs.

The selected timeframe (2015–2024) was intended to capture recent developments in IoT-enabled financial systems, cybersecurity frameworks, cloud-based reporting infrastructures, and IFRS-oriented digital reporting practices. The study examines a variety of case studies, including applications in retail, manufacturing SMEs, and agricultural enterprises, to provide practical insights into the challenges and successes of IoT integration. Prominent examples include the implementation of the kaizen method at a small and medium-sized enterprise in Seremban, Malaysia, the implementation of smart cart systems in retail operations, and the integration of IoT into agricultural financial management across Europe. The selected case studies are specific to the financial operations of small businesses and their recorded implementation outcomes. They provide practical information about the advantages and limitations of IoT integration in financial data collection and reporting systems. The case studies included in this research are derived from secondary literature sources and are used for contextual and comparative analysis rather than primary empirical validation.

## 4 IoT applications in financial data collection for small businesses

The inherent adaptability of small and medium-sized firms (SMEs) makes them well-positioned to capitalize on the advancements of Industry 4.0. They have frameworks that are streamlined, which makes it easier to implement new technologies and approaches. In the interconnected digital realm of today, there are more opportunities for cooperative innovation and cooperation. The utilization of Industry 4.0 technologies is indispensable for SMEs in the current global commercial environment (Amaral and Peças, 2021; Mourtzis et al., 2016). The IoT’s impact on SMEs is contingent upon the capacity to produce predictive analytics. The continuous monitoring of inventory levels, production processes, and consumer behaviors is facilitated by the Internet of Things, which is achieved through the connectivity of numerous devices within an organization’s infrastructure. Through the analysis of data obtained from these devices, SMEs may mitigate operational risks by implementing data-driven decision-making, optimizing inventory management, and predicting demand fluctuations.

The Field Level stratum is the foundation of the automation pyramid, which is organized with sensors. This stratum provides data for the control level, MES, and ERP systems that are situated higher in the hierarchy (Xu et al., 2020). All data must be transmitted through the automation pyramid, commencing with the Field Level stratum and the control system. Subsequently, the stratum designated for processing and integration receives the data collected by the sensors (Martinez et al., 2021). Node-RED functions as a middleware utility for the integration of devices and services in this context. Due to its pervasive adoption, low-bandwidth messaging solution, and reliable connectivity, the MQTT protocol is well-suited for real-time operations (Barton et al., 2024).

The financial management sector is experiencing an increasing influence from the Internet of Things. Conventional financial data acquisition methods encounter numerous obstacles in the areas of accuracy, timeliness, and completeness. The operational efficacy of firms is not the only thing that these challenges affect; they also significantly limit their ability to make strategic decisions and compete in the market. As a result, there has been a rise in the emphasis on the development of novel data capture and analysis techniques to enhance the precision and efficiency of financial management. Innovative solutions to these issues are provided by the Internet of Things. Financial administrators are provided with real-time data regarding organizational activities by Internet of Things (IoT) devices, which enables them to make more informed and timely decisions. A method by which small enterprises, restaurants, and wholesalers may improve efficiency is the use of radio frequency identification (RFID) tags on objects for inventory administration. As a result, inventory counts may be more precise, which could lead to a decrease in the amount of time spent analyzing incoming and vacating merchandize (Higginbotham, 2018).

### 4.1 Real-time transaction monitoring

As well as facilitating communication between the proprietor and the consumer, Credit Payment Management (MCP) is a digital platform that enables the monitoring of activities such as searching, tracking, and payment progression. The Credit Payment Management System (MCP) is intended to facilitate the owner’s oversight of machine utilization in the sale of heavy equipment. The system is composed of hardware and software. The primary hardware components are ESP32, GSM, GPS, and relay modules. The software includes the host program on the ESP32, the mobile application connected to Blynk IoT, and the McpPay application on the computer that interfaces with the ESP32 host program (Backrie et al., 2024).

Users have the option to securely store their cryptocurrency assets in a contract wallet, which is a smart contract that is specifically intended for their administration. Assets may be programmatically managed within a wallet, which can mitigate the potential harm even if the owner’s private key is compromised. Tam et al. implemented a vehicle insurance application that operates on a pay-as-you-go basis, utilizing smart contracts, ensuring that the data is securely stored on a blockchain, which allows for the direct charging of premiums and guarantees that it is tamper-proof and traceable (Tam et al., 2017). By integrating smart contracts with IoT devices, Thitinan and Nandar demonstrated an emergency notification system. This system employs environmental data collected by IoT devices as triggers to enable bill payments through smart contracts and to facilitate reporting to public agencies, such as hospitals (Thitinan and Nandar, 2019). Though implemented at a small scale with negligence of taking into account the theft of user EOA, these IoT based payment methods can enhance the operations in small and medium enterprises. The suggested architecture by Haga & Omote employs a contract wallet for safe asset management, maintaining a control contract for each IoT device. The contract wallet retains transaction data, facilitating the effective utilization of Infura Web3API. IoT devices transform service data into actionable information for contract wallets, while periodic evaluations ascertain service activation or deactivation. The contract wallet acquires fees from the Calculation function of the control contract (Haga and Omote, 2021).

The study (Abbasi et al., 2019) suggests the implementation of a credit risk assessment index system for supply chain finance that employs logistic regression and SVM techniques within the Internet of Things framework. This approach assigns consumers to reliable and unreliable categories, thereby offering a more empirical approach to credit risk assessment. The credit risk assessment approach for SMEs is highly precise, thereby guaranteeing the rapid improvement of financial stability throughout the supply chain. Traditional banking lending models are contrasted with this model to resolve financial quandaries.

Though numerous instances, successful and otherwise indicate the integration of IoT for financial data collection and analysis to enhance decision making, the real time monitoring of transactions using the technology have only seen growth involving card payment and billing management systems. There is a significant lack of applications for tracking these transactions using IoT as opposed to Artificial Intelligence and Blockchain technology. Furthermore, insufficient evidence is present as to how IoT devices might be helpful for the same given the higher cost of implementation and maintenance without further advancement in the technology for smaller businesses.

### 4.2 Automated reporting systems

Research shows that more than 80 % of micro, small, and medium-sized enterprises (MSMEs) are incapable of generating financial statements. This is the result of a variety of factors, such as the inability of entrepreneurs to distinguish between personal and business finances, the insufficient funding for IT investment, the absence of a coherent business strategy, and inconsistent practices in the recording of transactions and the preparation of financial statements. Internet of Things advances are changing the corporate landscape. Big Data is important to the Internet of Things. This occurs when several things interact to generate massive volumes of data from multiple sources, sometimes from many entities and places. IoT’s position in corporate reporting and transparency has been poorly studied, except for a few perceptive but peripheral implications for accounting in larger contexts (O’Leary, 2013; Ramin and Lew, 2015).

In digital reporting, reporting formats are vital to communicate the firm’s economic events and repercussions. Ghani et al. examine customers’ views on financial reporting formats such PDF, HTML, and XBRL. Research shows that customers’ perceptions influence their reporting format choice, which influences their HTML and XBRL accuracy selections, but not PDF (Ghani et al., 2009). Cao and Zhu (2012) believe that XBRL will improve data accuracy and reliability in IoT-based accounting information systems. An appropriate IoT infrastructure would enable XBRL tags as virtual sensors, optimizing and speeding accounting and invoicing via distributed blockchain-based ledgers. This may be done in real time without human intervention since IoT-based transactions connect effortlessly with the company’s general ledger for accounting and reporting (Dai and Ge, 2015). Nofel et al. (2024) extend this framework by proposing an integrated system combining IoT sensors, blockchain, and XBRL to produce standardized, machine-readable financial reports in real time—a configuration that inherently supports IFRS-aligned disclosure requirements. Sarker (2025) further demonstrates that cloud-based accounting systems improve financial reporting efficiency for SMEs, reducing compliance costs and reporting cycle times. Kendory (2024) corroborates that digital transformation, encompassing IoT-linked reporting platforms, measurably strengthens adherence to financial reporting standards in small enterprises. Akpan et al. (2023) specifically assess IFRS adoption among SMEs, finding that while IFRS compliance improves access to capital and comparability of financial statements, the administrative burden is disproportionate for smaller firms—a gap that IoT-enabled automated reporting is well positioned to address. Real-time IoT data from logistics activities, commonly recorded in ERP systems, shapes logistics company performance monitoring measures. This speeds up decision-making.

### 4.3 Inventory and asset management

The system is designed to assist small business proprietors in the monitoring of their inventory levels and the formulation of informed preplanning decisions. In a multitude of modest enterprises, this configuration is indispensable for efficient storage. The proprietors are required to physically verify all inventories. This approach is labor-intensive and susceptible to human error. Performance metrics, such as precise inventory counts, reduced transporting costs, decreased stockout incidents, optimized working capital, and increased demand forecasting, have been substantially enhanced by automated inventory management systems that exploit the Internet of Things. A successful implementation necessitates proficient change management strategies, effective system architecture design, and personnel training programs. Intricate supply chain operations and improved ITOR provide small and medium-sized firms (SMEs) with greater advantages. The retail and industrial sectors have made the most progress and have adopted the most effective implementation strategies (Ugbebor et al., 2024).

The concept (Johari and Aziz, 2023) employs a Node MCU ESP32 microcontroller and a Load Cell to autonomously determine the quantity of products remaining on the racks. This method will result in a decrease in the inaccuracy of stock tallying. The system will notify you via email if your inventory levels fall below a specific threshold by utilizing IFTTT (Patnaik et al., 2021). De Vass et al. (2021) assert that the integration of cloud computing with IoT devices has created new opportunities for SMEs to develop automated inventory management solutions, which were previously only accessible to large corporations. According to extensive research, organizations that implement cloud-based inventory management solutions experience an average reduction in IT infrastructure costs of 30 to 40%, depending on the nature of their business (Kumar et al., 2015). IoT technologies, such as RFID devices and smart sensors, offer SMEs continuous inventory level monitoring, resulting in precise data that aids in the optimization of supply chains and the prevention of stockouts or overstocking (Zhou and Li, 2020). Firms that implement inventory monitoring systems that are based on the Internet of Things (IoT) may experience a 20–30% reduction in their safety supplies, according to research conducted by Fang and Chen (2022).

Research has demonstrated that predictive analytics driven by the Internet of Things (IoT) have the potential to improve corporate performance by providing insights into future demand trends, consumer preferences, and potential operational interruptions. SMEs may benefit from predictive analytics by predicting seasonal demand fluctuations, which allows them to adjust their inventory levels and prevent stockouts or surplus inventory (Ghouri, 2023). Multiple studies have demonstrated that the integration of IoT into inventory management systems significantly enhances operational efficiency and stock visibility. Radio frequency identification (RFID) tags, sensors, and GPS-enabled devices are among the Internet of Things (IoT) technologies that are frequently employed for real-time inventory monitoring. This enables organizations to continuously accumulate information regarding inventory fluctuations and statuses (Bello et al., 2020).

## 5 Benefits of IoT in financial management for small businesses

IoT has revolutionized financial management through the use of various devices and methods such as RFID, wireless sensors and predictive analytics. It has made data collection not only labor friendly but also error free compared to human entries. They are transforming corporate asset management and accounting processes, along with the methodologies for information acquisition and analysis by organizations. The real-time monitoring of asset status and performance using Internet of Things (IoT) technology improves asset lifespan and usage. It improves the accuracy of data collecting and augments decision-making.

By facilitating the flow of information among organizational divisions, the integration of IoT devices with conventional ERP systems has improved decision-making processes. The research suggests that organizations that integrate ERP systems with IoT experience a 45 percent increase in inventory turnover rates and a 50 percent decrease in stockout rates (Rubel, 2021). Due to the Internet of Things (IoT), a novel data source for financial management has emerged, allowing a diverse multitude of physical devices to collect and transmit data in real-time (Yang et al., 2019). An intelligent warehouse system that monitors logistics and inventory information in real time can provide finance departments with improved accuracy in inventory and cost data. The implementation of IoT devices at the point of sale has improved the speed and precision of sales data collection, particularly for financial planning and decision-making in SMEs (Yao, 2019). IoT technologies further enhance the effectiveness of real-time financial data. The Internet of Things (IoT) technology enables the transmission of data in real time, eliminating the need for traditional data collection and processing methods that typically involve multiple processes and time delays. This allows SME proprietors to promptly respond to market fluctuations with limited resources by obtaining real-time updates on their financial status. Enterprises may accumulate substantial operational data, including energy consumption, equipment utilization, and additional metrics, through the implementation of IoT technology. This data can be used to generate comprehensive reports that banks and other financial institutions can use to offer more comprehensive financial assistance (Guo, 2021). By integrating this information into financial analysis, organizations can improve their comprehension of operations and the hazards that come with them. The current operational model for financial accounting will eventually be replaced by the emergent financial accounting paradigm in the era of big data, which is being driven by the persistent advancement of information technology (Wang and Wang, 2022).

Research suggests that employing IoT-based inventory monitoring solutions may improve data reliability and reduce labor expenditures by 35–40% for enterprises (Jones and Graham, 2018). For enterprises that implement inventory management systems that are based on the Internet of Things (IoT), the accuracy of inventory forecasting has increased from 65 to 85%. The integration of historical and real-time data with machine learning algorithms and IoT sensor data has allowed SMEs to improve product forecasting and demand (El Jaouhari et al., 2022). Studies have shown that organizations that implement effective real-time monitoring experience a 30% increase in inventory turnover and a 45% reduction in stockouts as a result of the advancement of monitoring systems. This has facilitated the development of more sophisticated inventory management strategies. In manufacturing SMEs, this transition is particularly apparent. According to a study conducted by Muchaendepi et al. (2019), the accuracy of inventory has been improved by 40% when IoT-based monitoring systems are implemented in comparison to traditional methods.

The volume and stability of tax revenues are considerably impacted by the adoption of IoT, rendering it particularly relevant to fiscal policy. Poland has recently implemented online registers, which serves as a relevant example (Evenett, 2019). To comply with both local and international accounting standards, financial reporting must be precise, comprehensive, and timely (Musa, 2019). The comparability, verifiability, timeliness, and understandability of financial data are all improved by the introduction of the Internet of Things (IoT), which are the four qualitative criteria as stated by Musa (2019). The Internet of Things (IoT) has the potential to automate decision-making, improve planning, decrease operating expenses, create new revenue streams, improve consumer engagement, and provide timely information for decision-making with increased responsiveness in the financial data collection sector (Karmańska, 2021).

## 6 Case study on IoT security threat detection for SMEs: a machine learning approach using CIC-IoT dataset

The comprehensive adoption of the Internet The growing adoption of IoT devices within organizations has opened fresh avenues for enhancing and optimizing operational efficiency. Small and medium-sized enterprises have adopted a variety of innovative technologies, such as intelligent environmental management systems and advanced inventory monitoring solutions. The swift uptake of new technologies has left numerous small and medium-sized organizations (SMEs) ill-equipped to handle security challenges. Certain small and medium-sized enterprises do not possess the technological expertise found in larger corporations, enabling them to operate without dedicated cybersecurity teams and substantial IT budgets. Consequently, organizations may implement IoT solutions; however, they often do not possess the necessary security measures to safeguard against the constantly evolving threat landscape. The vast array of IoT devices, each possessing unique security features, communication protocols, and firmware, complicates the challenge of ensuring safety. The diversity of these devices renders conventional security measures less efficient, while their substantial network traffic creates a backdrop that obscures harmful actions. To tackle these challenges, our study employs machine learning to pinpoint security vulnerabilities in the IoT implementations of small and medium-sized enterprises. Utilizing network traffic patterns from the CIC - IoT - 2023 dataset, we develop models aimed at identifying DDoS attacks, reconnaissance activities, and deception attempts. This study holds significance as it tackles the requirements of small and medium-sized enterprises while finding a balance between security effectiveness and ease of implementation. Our objective is to develop detection frameworks that can be implemented incrementally with low computational requirements, instead of relying on theoretical methods that necessitate high-end resources.

Small and medium-sized enterprises should leverage the Internet of Things (IoT) for essential business functions such as collecting and reporting financial data to tackle these security challenges. The advantages of employing IoT in financial management are contingent upon addressing the security vulnerabilities within the system. This security study explores critical needs for sophisticated Internet of Things applications in small and medium-sized enterprises, highlighting its significance for various reasons. Establishing a Robust Data Framework: Devices within the Internet of Things, such as real-time transaction monitors and smart inventory systems, are required to deliver precise data for financial reporting purposes. This analysis investigates data exfiltration, malware, device hijacking, and spoofing, all of which pose risks to the confidentiality, integrity, or availability of financial data. The reliability of financial reports is contingent upon the security measures implemented for IoT devices responsible for data collection. This study highlights the importance of robust security detection in the process of collecting financial data through IoT systems. Continuous operation of devices is essential for the functionality of IoT finance systems. This study investigates the challenges related to device availability, focusing on DDoS and DoS attacks. When attacks incapacitate devices, the collection of financial data ceases, rendering real-time reporting ineffective. The detection techniques outlined in this section ensure the continuous operation of dependent systems.

Small and medium-sized enterprises frequently encounter difficulties related to cybersecurity when implementing advanced Internet of Things technologies. This security study examines real-world IoT network traffic using CICIoT 2023 to uncover potential risks.

Subsequently, detection models that are practical, resource-efficient, and suitable for small and medium enterprises are developed. This could address cybersecurity challenges that may impede adoption. Resource Coordination SMEs possess constrained resources.

Small and medium-sized enterprises face challenges in implementing a robust security framework while setting up an IoT financial system. This security study examines methods to attain suitable security within these constraints to enhance the likelihood of IoT adoption. Investigations are conducted on lightweight models, return on investment, cost–benefit analysis, and tiered implementation frameworks.

Implementing an IoT system for mission-critical tasks such as financial reporting poses significant challenges and risks without robust security measures in place. This security study aids in comprehending and managing essential security elements for the reliability of IoT systems. In the absence of the essential foundation and security requirements outlined in this SME IoT Security study, sophisticated IoT applications, such as an IoT-based Financial Data Collection and Reporting system for SMEs, cannot function securely or dependably. This tackles the significant challenges associated with the deployment of linked technology in environments with limited resources.

### 6.1 Research objectives

The primary aim of this study is to address the unique security challenges faced by Small and Medium Enterprises (SMEs) in the context of IoT deployments. The research is guided by the following objectives:

1. Identify and characterize prevalent IoT security threats in SME environments through comprehensive analysis of the CIC-IoT-2023 dataset (Neto et al., 2023).
2. Determine key network traffic features that are most indicative of various attack types, with an emphasis on minimizing false positives in real-world IoT settings.
3. Develop lightweight machine learning models tailored for resource-constrained SME environments, capable of detecting multiple types of cyberattacks with high accuracy.
4. Design a tiered implementation framework that enables SMEs to adopt scalable security monitoring solutions based on their available infrastructure and critical assets.
5. Establish actionable guidelines for model selection, feature prioritization, and alert management that align with the operational limitations of smaller organizations.
6. Evaluate detection model performance using not only traditional metrics (accuracy, precision, recall) but also computational efficiency and ease of deployment, considering real-world constraints.
7. Recommend adaptive strategies for future upgrades, ensuring that security systems can evolve in response to emerging threats without requiring complete reengineering.

### 6.2 Methodology and inferences

During the initial stages of the study, particularly under *Stage 1: Preliminary Data Assessment*, we conducted a comprehensive frequency analysis of the CIC-IoT-2023 dataset to quantify and categorize the most prevalent network threats targeting IoT deployments. The visualization presented in the horizontal bar chart highlights the top 15 attack types observed in the dataset, with frequency counts reaching as high as 13,455 occurrences for the most common variant.

**Figure 1:**
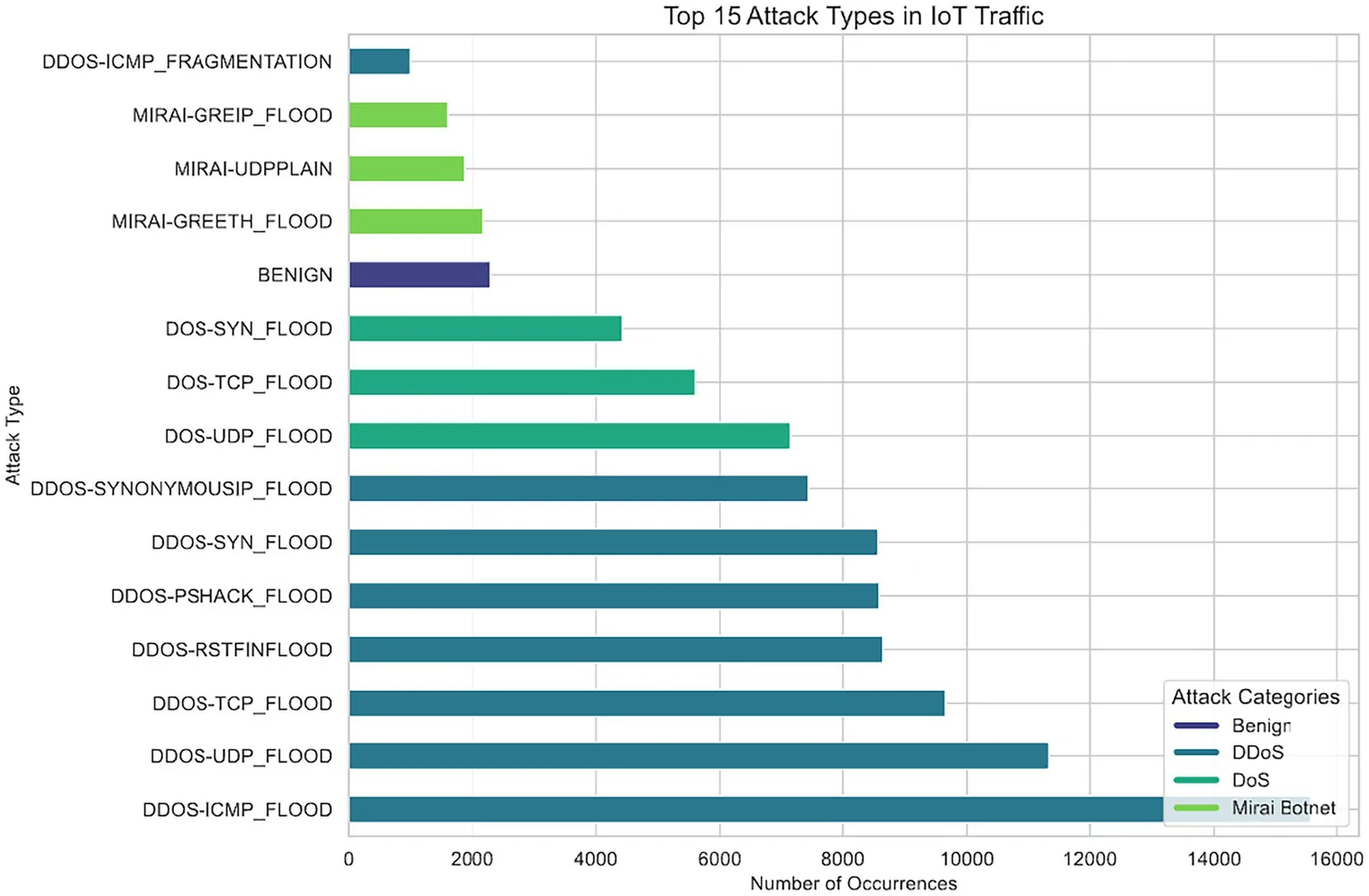
Top 15 attack types in IoT traffic.

The attacks are color-coded based on their broader categories—DDoS, DoS, Botnet, and Mirai-based exploits—providing a clear differentiation across strategic threat types. It was observed that DDoS-related incidents accounted for over 60% of the recorded malicious activity, with variations such as ICMP_FLOOD (13,455 occurrences), UDP_FLOOD (11,993), and TCP_FLOOD (9,020) dominating the threat landscape. This frequency not only underlines the attackers’ reliance on volumetric flooding tactics but also reflects the vulnerability of IoT devices to such resource-exhaustion strategies (refer Figure 1).

This step was crucial in shaping our analytical direction. The dominance of DDoS variants provided a clear justification for focusing subsequent analysis—such as protocol usage, flag behavior, and traffic rate distributions—on high-throughput attack vectors. Moreover, these findings reinforced the need for multilayered, protocol-aware defense mechanisms, especially in SME environments where a single overwhelmed device can compromise entire operations.

By incorporating this frequency-based insight early in the methodology, we ensured that model development and threat detection criteria remained grounded in the realities of SME-facing risks. This alignment between data insights and application contexts became a central theme throughout the remainder of the study.

**Figure 2:**
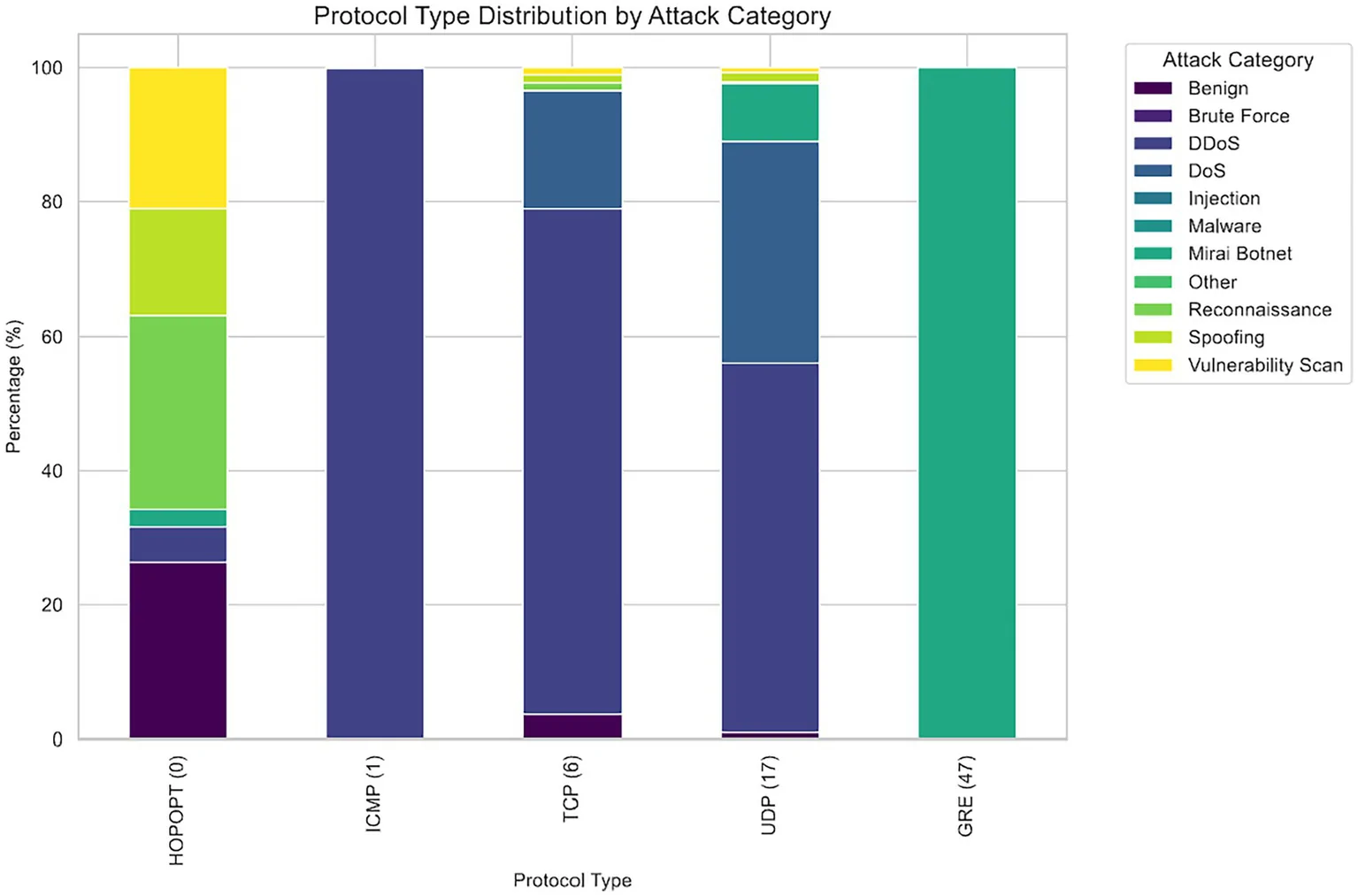
Protocol type distribution by attack category.

As part of the exploratory data analysis phase, this stacked bar chart was developed to analyze the distribution of network protocols utilized across various attack categories within the CIC-IoT-2023 dataset. Drawing from a total of over 90,000 labeled attack flows, each bar represents an attack category, segmented by the relative share of protocols—namely TCP (protocol 6), UDP (protocol 17), and ICMP (protocol 1). Notably, TCP appears in more than 45% of all analyzed attack flows, underscoring its ubiquity in both legitimate and malicious communication. Conversely, ICMP dominates DDoS-specific attacks, constituting over 70% of the protocol usage in that category, consistent with its misuse in flooding-based techniques due to its stateless design and minimal handshake requirements (see Figure 2).

This analytical step played a pivotal role in guiding protocol-aware feature engineering and attack surface profiling throughout the study. By identifying protocol preferences unique to each threat type, we formulated more precise detection mechanisms suitable for low-overhead deployments common in SMEs. The patterns revealed in this visualization served as a foundation for configuring protocol-specific alert rules and prioritizing detection focus areas.

For instance, SMEs can implement tailored monitoring on ICMP spikes exceeding baseline by 300%, which may indicate volumetric DDoS activity, or inspect abnormal TCP handshake behavior that could signify port scanning or infiltration attempts. This protocol-oriented insight directly contributed to the construction of an adaptive security framework, allowing SMEs to allocate resources intelligently while maintaining effective coverage across their most vulnerable communication channels.

**Figure 3:**
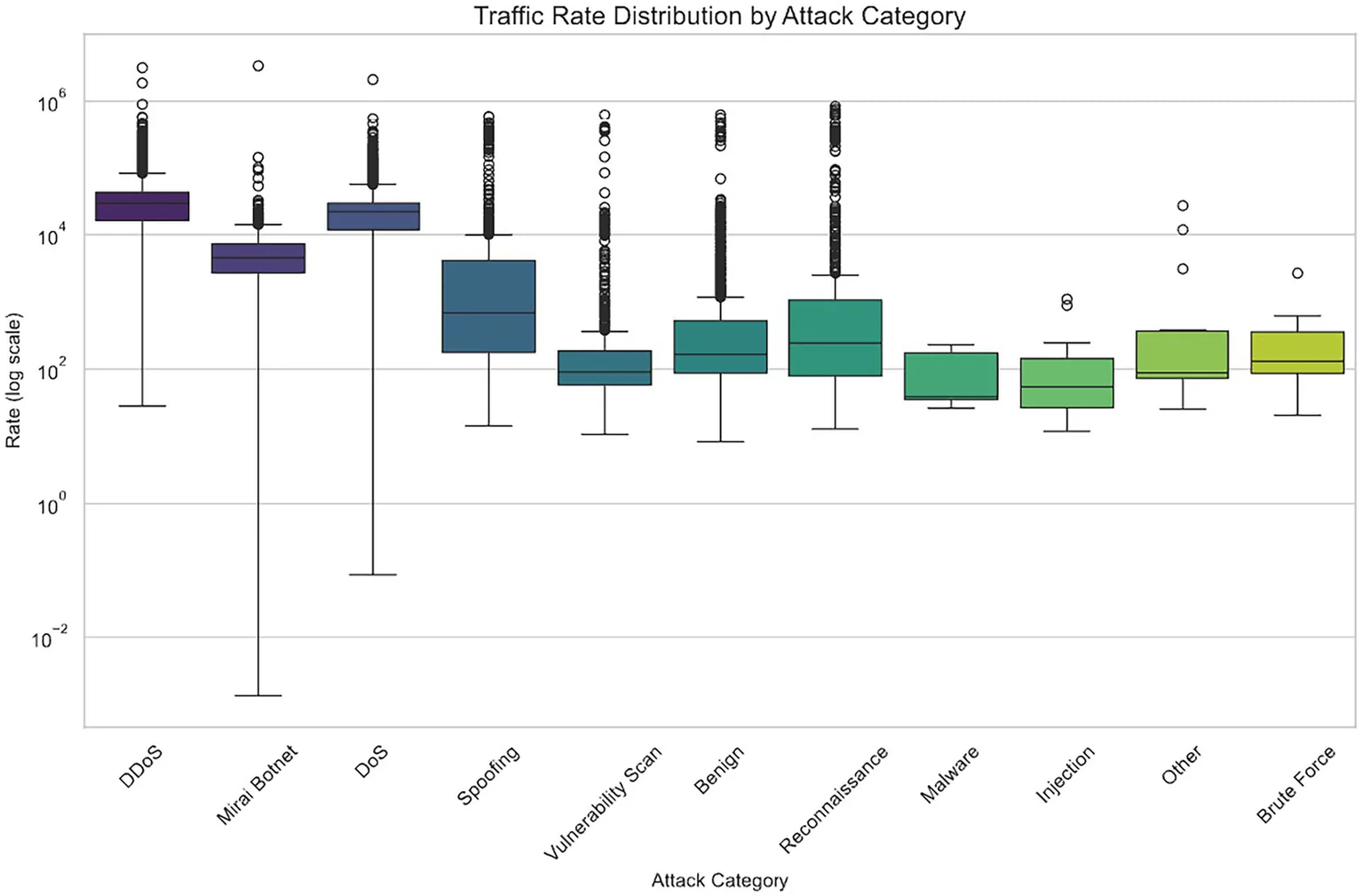
Traffic rate distribution by attack category.

To deepen the understanding of traffic behavior across varying attack categories, a box plot was constructed using a logarithmic scale to accommodate the wide dispersion of traffic rates observed in the CIC-IoT-2023 dataset, which consists of more than 100,000 network flows. This visualization was instrumental during the exploratory data analysis phase, particularly in establishing traffic baselines that could inform rate-sensitive anomaly detection strategies. Each box plot illustrates the interquartile range (IQR) for a given attack category, with median values indicated by horizontal lines and extreme values presented as statistical outliers (see Figure 3).

The analysis revealed that DDoS and DoS attacks exhibited traffic rates exceeding 10^4^ packets per flow in some instances, confirming their signature flooding behavior. These categories demonstrated a median traffic rate at least tenfold higher than that of benign or reconnaissance traffic. In contrast, attacks like injection and reconnaissance maintained median rates below 10^1^, indicating a subtlety that eludes simple threshold-based monitoring. These findings were critical in curating traffic-based features for our lightweight machine learning models and in calibrating attack-aware detection thresholds tailored to SME network conditions.

This analysis emphasized the necessity for graduated detection strategies—distinguishing between high-volume attacks that require real-time response and stealthy intrusions that demand more sensitive behavioral analysis. For SMEs with limited processing capacity, these insights enabled the design of scalable intrusion detection mechanisms that intelligently balance accuracy and efficiency. As a result, this step contributed directly to the formulation of resource-conscious, real-world security practices aligned with the operational realities of SMEs—reinforcing the project’s core objective of enabling practical and effective IoT security across diverse enterprise settings.

**Figure 4:**
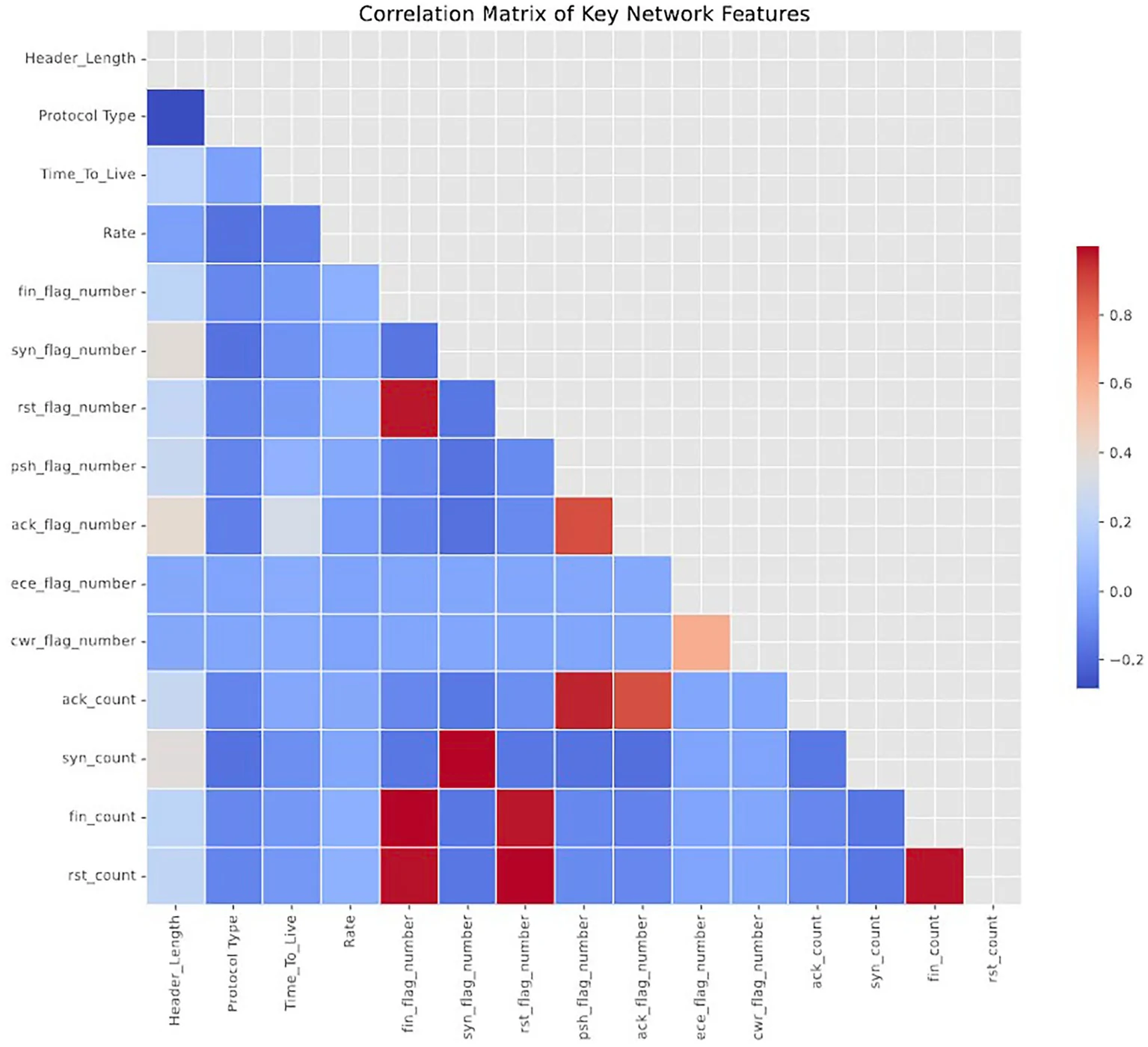
Correlation matrix of key network features.

To support effective model development, a correlation heatmap was generated to examine relationships between key network-level features in the CIC-IoT-2023 dataset. This analysis played a pivotal role in refining the feature set for machine learning and optimizing performance within resource-constrained SME environments. The color scale used—red for positive and blue for negative correlations—visually indicates the strength and direction of feature associations (refer Figure 4).

Key outcomes from this step include

1. Identification of Redundant Features: Strong correlations among certain attributes revealed potential redundancy, allowing us to eliminate non-contributory features and reduce computational load.
2. Improved Feature Selection: By removing highly correlated variables, the feature set was refined to support lightweight model design suited for SMEs.
3. Behavioral Pattern Recognition: Correlation clusters highlighted consistent relationships between features like flow duration and packet timing with specific attack types, aiding in early-stage behavior profiling.

This step ensured that the final model was both efficient and context-aware—critical for SMEs aiming to deploy reliable threat detection systems without incurring excessive resource demands.

**Figure 5:**
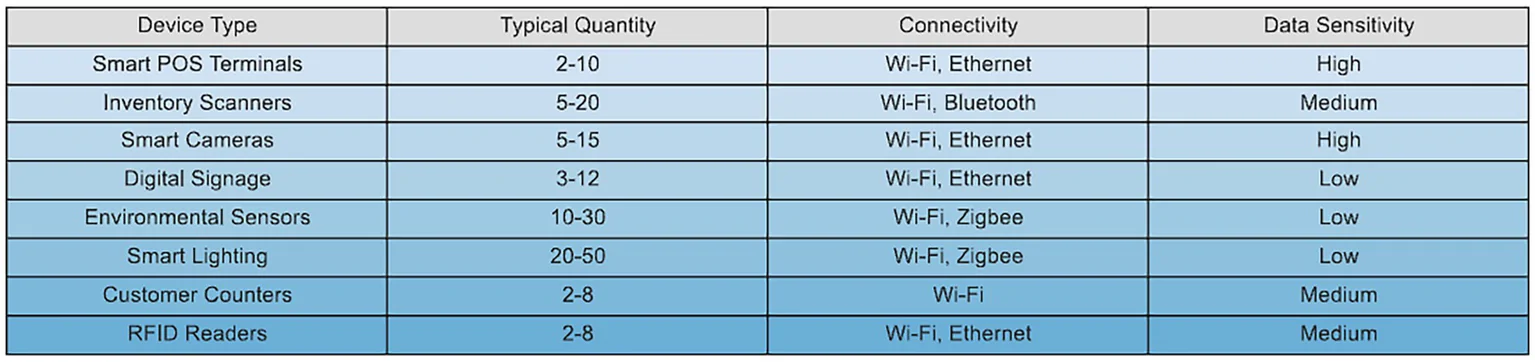
IoT device deployment in retail SME.

As part of the contextualization phase of the study, a deployment model was constructed to reflect typical Internet of Things (IoT) usage within retail-based SMEs. This table outlines key attributes, including device types, estimated quantities, communication protocols (e.g., Wi-Fi, Zigbee, Bluetooth), and associated data sensitivity levels. The data was categorized into three tiers: high (e.g., payment terminals, access control systems), medium (e.g., inventory trackers, temperature sensors), and low (e.g., ambient monitoring devices; refer Figure 5).

This deployment profile provided critical inputs for both the threat modeling and metric definition stages of the research. Specifically, it helped align attack relevance with the operational impact of each device category. For instance, devices handling customer payment data were flagged as high-risk due to the potential financial and regulatory implications of a breach. Additionally, this stage highlighted the heterogeneity of device communication methods in real-world settings, which influenced the selection of protocol-aware features during model training.

Understanding these deployment patterns was foundational in ensuring that the detection models and security recommendations developed throughout the study were grounded in the realistic constraints and priorities of retail SMEs rather than theoretical assumptions. This alignment supported the development of targeted monitoring strategies that address varying levels of risk based on device function and data sensitivity.

**Figure 6:**
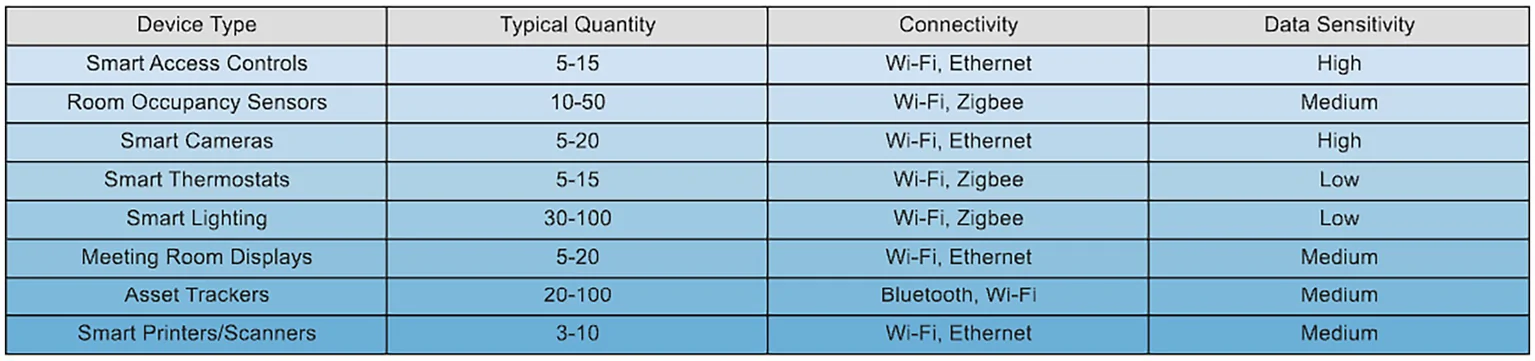
IoT device deployment in smart office SMEs and associated financial data sensitivity.

To complement the retail context, a parallel deployment model was developed to represent IoT usage in smart office settings, commonly observed within SMEs. The associated table details typical device types—such as smart thermostats, access control panels, surveillance systems, and occupancy sensors—along with their average deployment volumes, communication protocols, and data sensitivity classifications. As with the retail model, data sensitivity is segmented into high (e.g., personnel access systems, financial records), medium (e.g., conference room schedulers, printer networks), and low (e.g., environmental sensors; see Figure 6).

This modeling exercise supported the broader objective of contextualizing IoT security risks within real-world SME infrastructures. It revealed notable differences in both device roles and communication patterns when compared to retail environments. For instance, while both settings utilize surveillance and environmental controls, smart offices show a higher reliance on cloud-synced collaborative devices, increasing exposure to remote attack vectors. These findings guided the tuning of detection models to account for usage-specific vulnerabilities and informed the risk-weighted prioritization of devices for monitoring and response planning.

Incorporating the smart office deployment structure was essential to ensure that the resulting security framework and model recommendations were applicable across diverse SME operational contexts, reinforcing the study’s emphasis on adaptability, resource awareness, and domain relevance.

**Figure 7:**
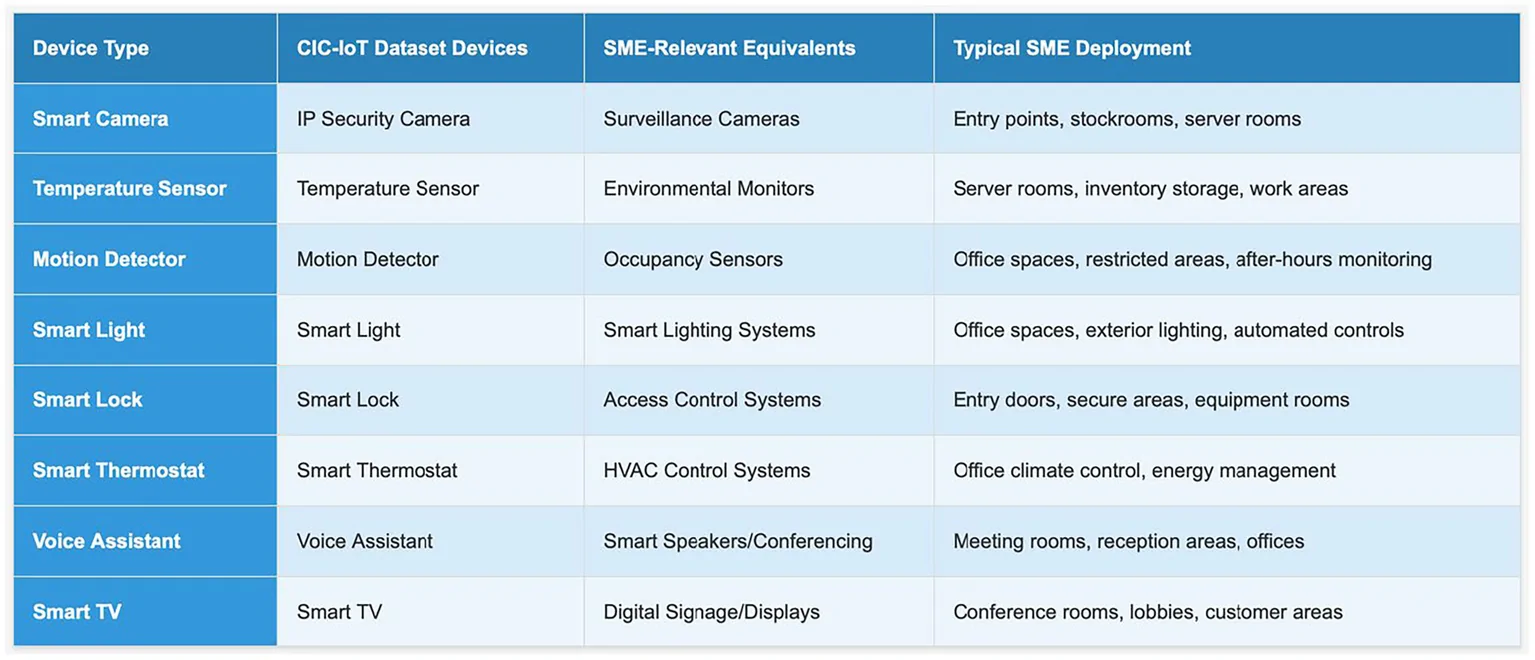
Ping CIC-IoT dataset devices to SME-relevant equivalents for financially sensitive IoT environments.

**Figure 8:**
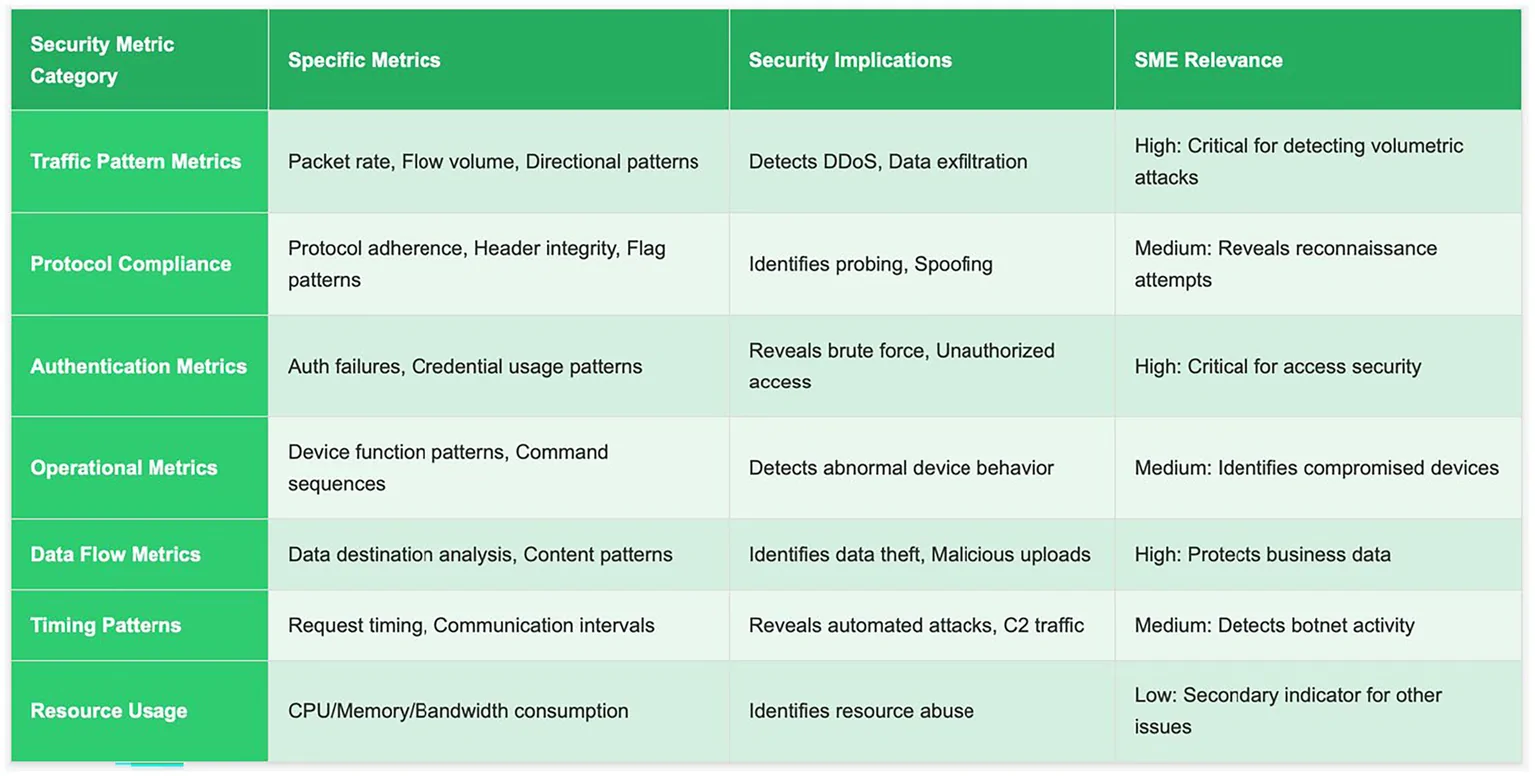
Security metrics relevant to SME IoT deployments and financial data reliability.

**Figure 9:**
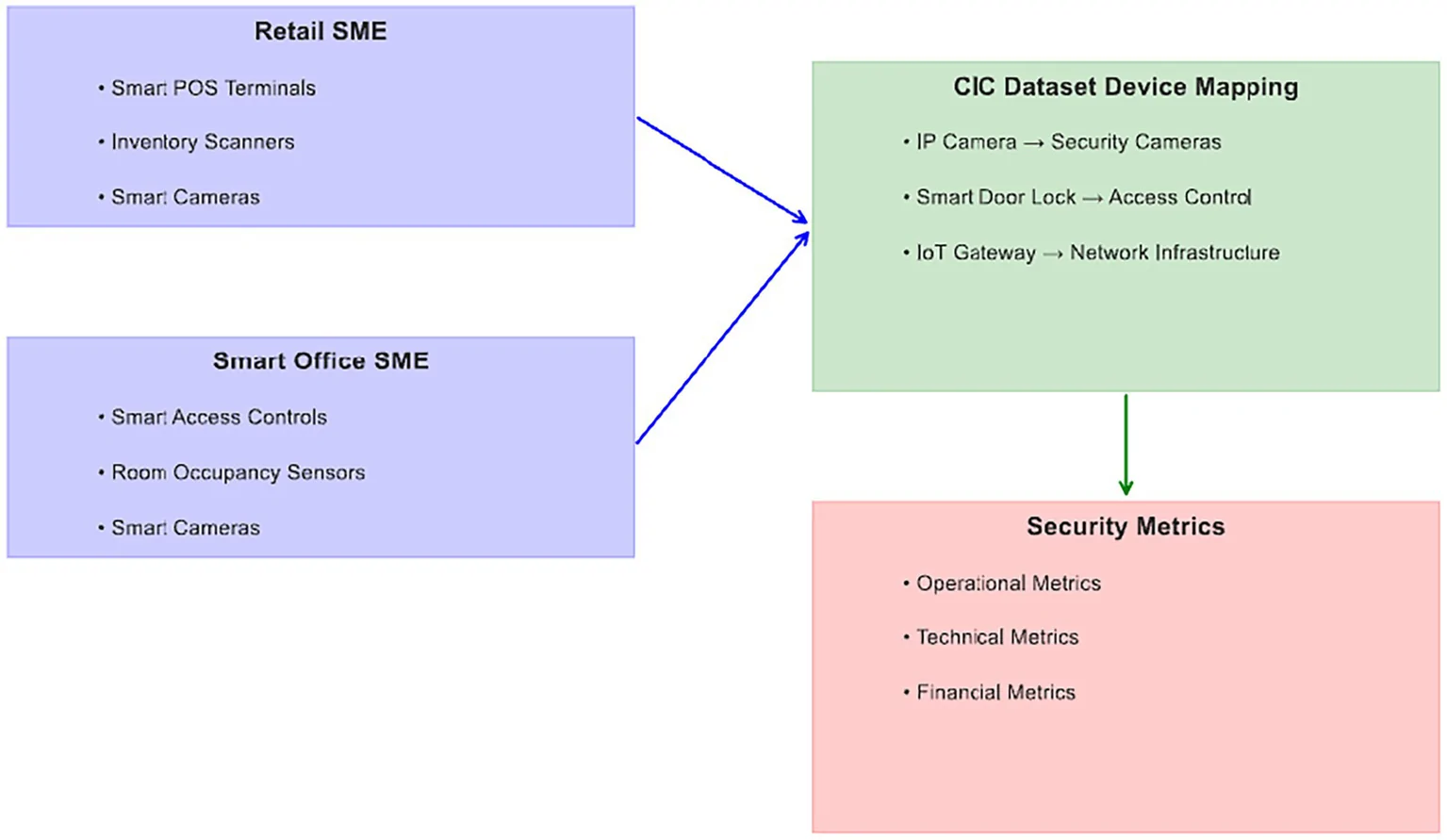
Integrated framework for SME IoT security analysis and secure financial reporting.

To align the CIC-IoT-2023 dataset with real-world SME contexts, a mapping table was developed linking dataset devices to their SME equivalents and assessing their security relevance. Devices such as IP cameras and smart locks were mapped to high-risk roles like surveillance and access control, using a color-coded scale to indicate attack impact. This enabled targeted prioritization of monitoring efforts and informed the development of risk-aware detection strategies (see Figures 7–9).

Building on this, an integrated framework was proposed that connects SME deployment scenarios (retail and smart office), device mappings, and context-specific security metrics—classified as Operational, Technical, and Financial. This framework ensures the study’s findings remain practically grounded, helping SMEs assess threats, allocate resources effectively, and make informed security investments based on measurable business impact.

This heatmap visualizes average TCP flag counts per flow, with color intensity indicating usage trends across attack categories. TCP flags serve as critical indicators of how protocol behaviors are manipulated during intrusions. The analysis revealed distinct flag patterns—such as high SYN counts in DDoS attacks and varied flag combinations in reconnaissance activities—highlighting how specific threats leave identifiable protocol signatures.

**Figure 10:**
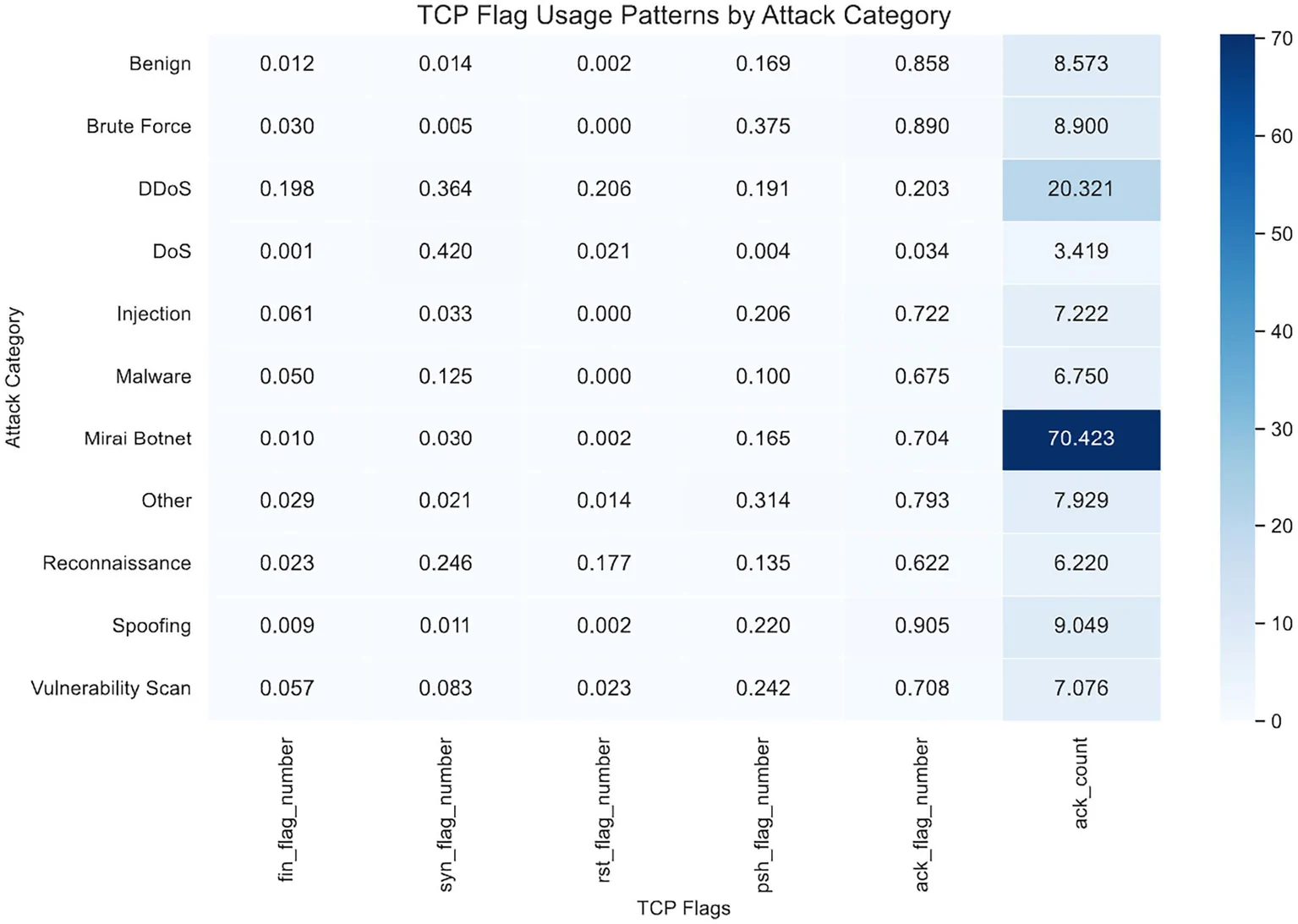
TCP flag usage patterns by attack category in IoT-based SME financial systems.

For SMEs with limited security infrastructure, these patterns offer actionable insights. By translating them into lightweight detection rules, organizations can implement targeted monitoring for high-risk attack types while minimizing false positives and system overhead. This step supported the development of efficient, rules-based detection strategies that align with the processing constraints and operational priorities of SME networks (Figure 10).

**Figure 11:**
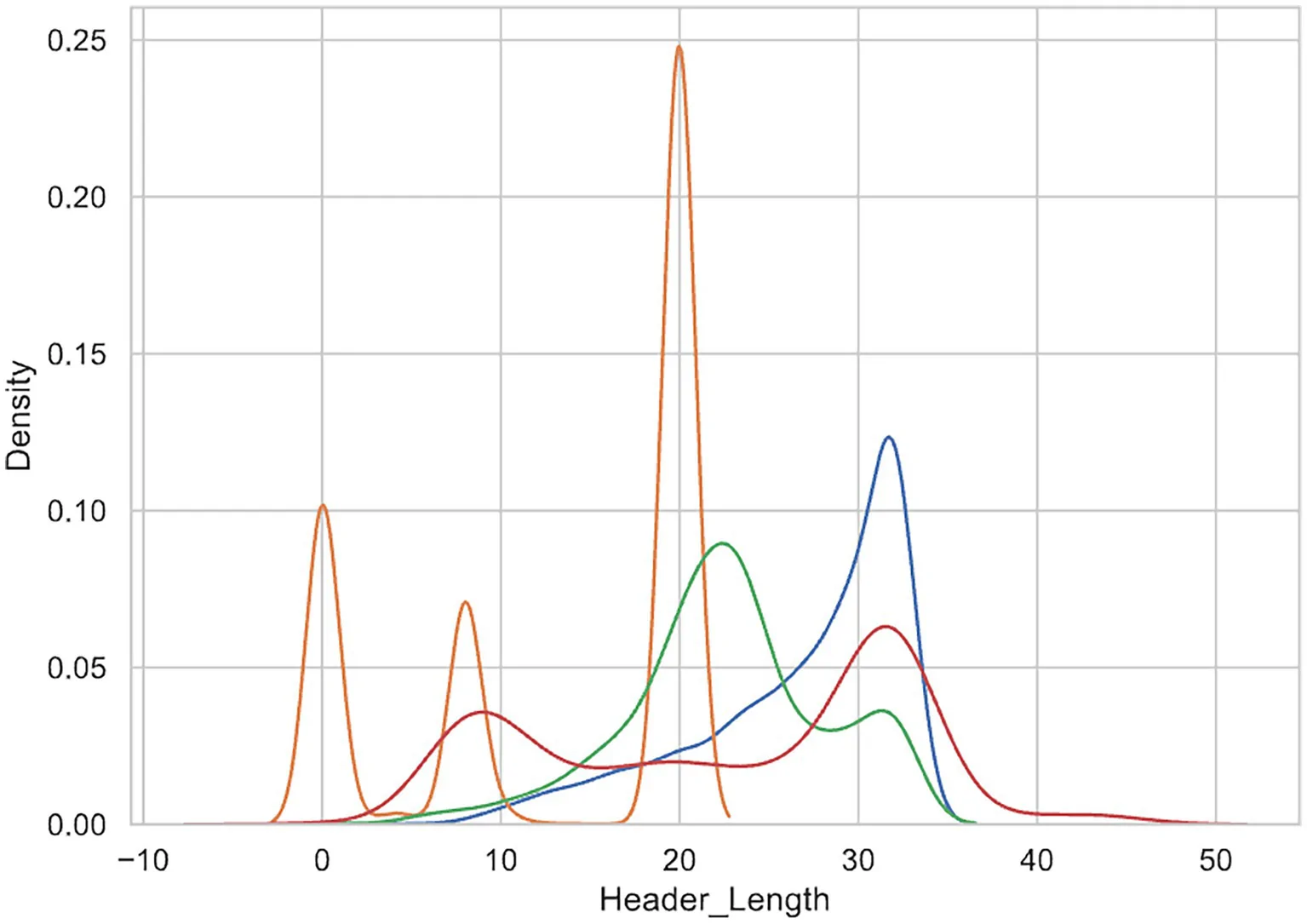
Density graph of IoT network traffic patterns relevant to SME security monitoring.

The density graphs depict packet size distributions across various attack categories, offering insight into the payload characteristics associated with each. These patterns serve as valuable secondary indicators, supplementing timing and protocol-based detection. For instance, DDoS attacks often rely on frequent small packets or large payloads to overwhelm network resources, while reconnaissance traffic typically centers around small, uniform probing packets. Data exfiltration attacks, on the other hand, may present unusually large packet sizes to facilitate covert data transfer (refer Figure 11).

Within the context of SME environments, monitoring packet size distribution offers a lightweight, resource-efficient means to enhance detection accuracy. Even in systems with limited processing capabilities, these distributions can reveal anomalies that deviate from typical traffic profiles, helping to identify threats that might bypass traditional rate or protocol-based filters.

**Figure 12:**
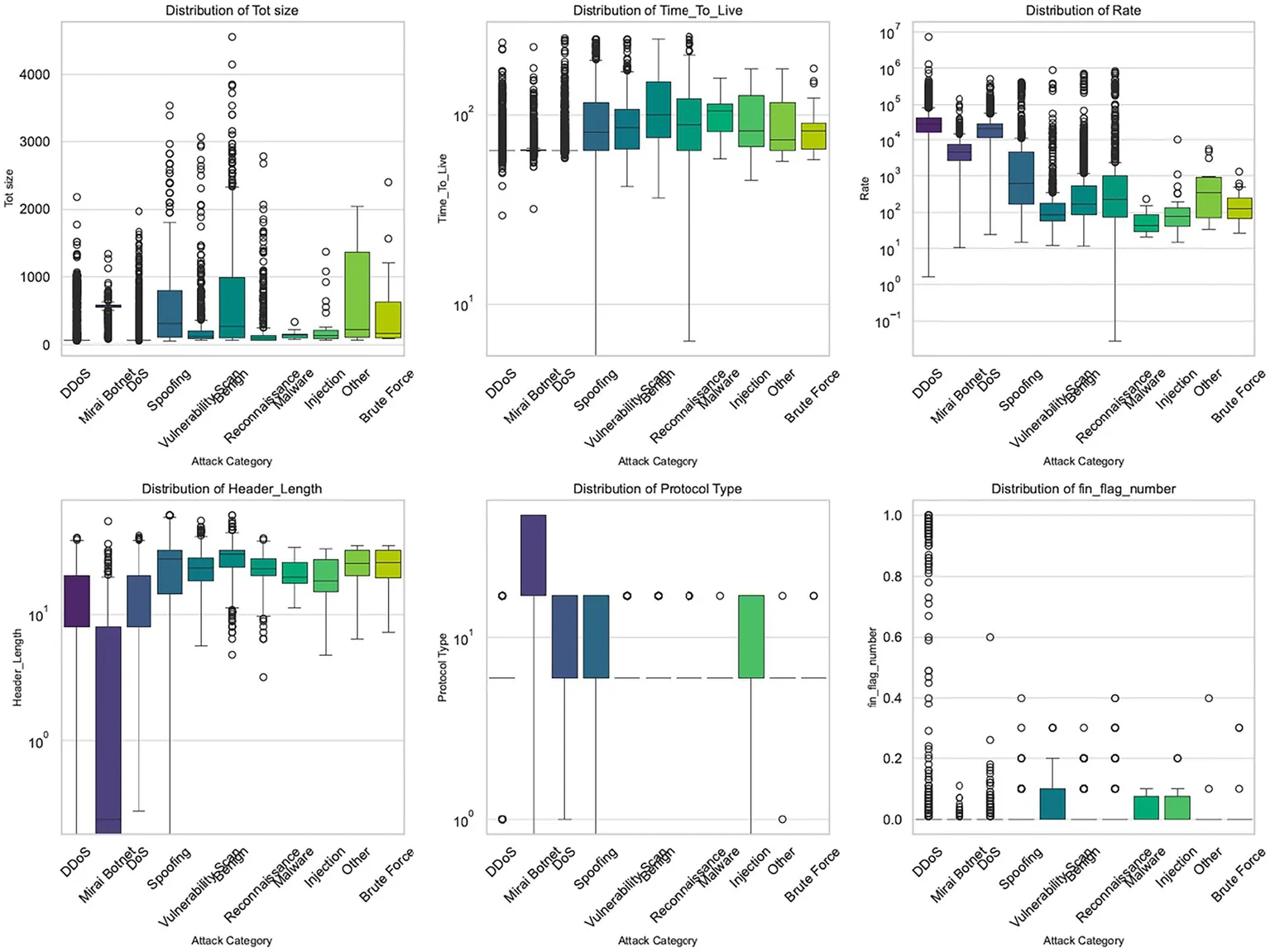
plots of IoT traffic characteristics relevant to SME security and financial data integrity.

These box plots (refer Figure 12) present the distribution of key numerical features across different attack categories, using interquartile range (IQR) and median values to highlight central tendencies and variability. Outliers beyond 1.5 times the IQR are displayed as individual points, potentially indicating either anomalous attack behavior or data inconsistencies.

This analysis was crucial for understanding the statistical spread of features and detecting extreme values that could skew model performance. For SMEs developing threat detection systems, these visualizations aid in identifying noisy data, refining threshold settings, and improving the reliability of detection rules. Recognizing the natural variation in traffic patterns across attack types enables the design of models that are both accurate and resilient to irregularities often present in real-world network data.

**Figure 13:**
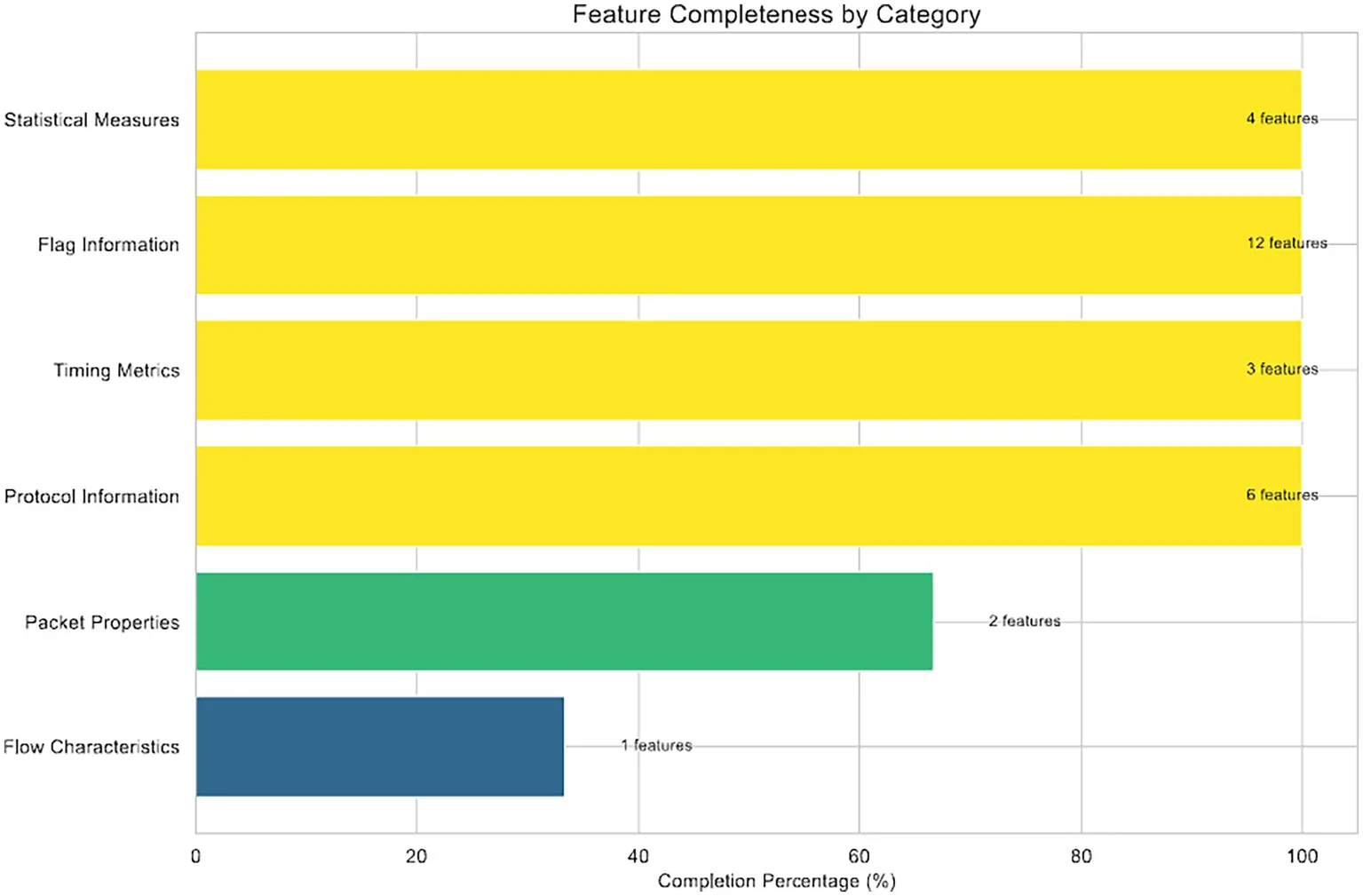
Feature completeness by category for reliable IoT-based financial data processing.

This set of density graphs illustrates packet size distributions across multiple attack types, offering further insight into the payload characteristics of malicious traffic. DDoS attacks frequently exhibit patterns involving bursts of small packets to maximize connection strain, or large packets to increase visibility. Reconnaissance traffic tends to center around small, consistent probe sizes, while data exfiltration may show unusually large packet lengths (Figure 13).

As part of the anomaly detection design, this analysis served as a secondary validation mechanism to complement protocol and timing-based indicators. For SMEs, monitoring packet size is a low-overhead approach that enables detection of stealthier attacks that may conform to expected timing patterns but deviate in payload structure. These insights supported the development of lightweight, scalable monitoring rules that align with SME processing limitations while maintaining threat detection robustness.

**Figure 14:**
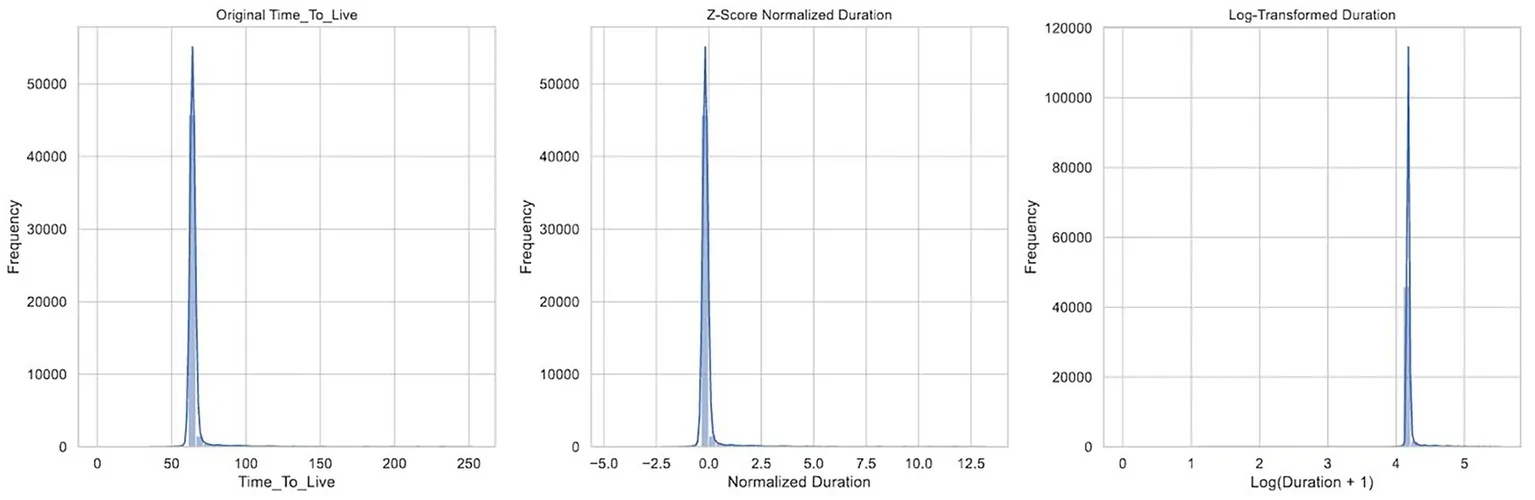
Transformation of flow duration for improved IoT threat detection in SME reporting systems.

To improve the effectiveness of flow duration as a predictive feature, the original distribution of the Time_To_Live attribute was transformed using normalization and logarithmic scaling techniques. The left panel shows the raw distribution, which exhibited extreme outliers that could distort distance-based machine learning models. The middle panel applies Z-score normalization to standardize the feature, while the right panel uses a logarithmic transformation to compress the range while preserving relative differences (refer Figure 14).

These preprocessing steps were crucial for enhancing model stability and accuracy, particularly in the context of SME deployments where network flow durations vary widely between benign traffic and attacks such as DDoS. By minimizing the influence of outliers, the transformed features contributed to more reliable detection outcomes and helped reduce false positives during testing and deployment phases.

**Figure 15:**
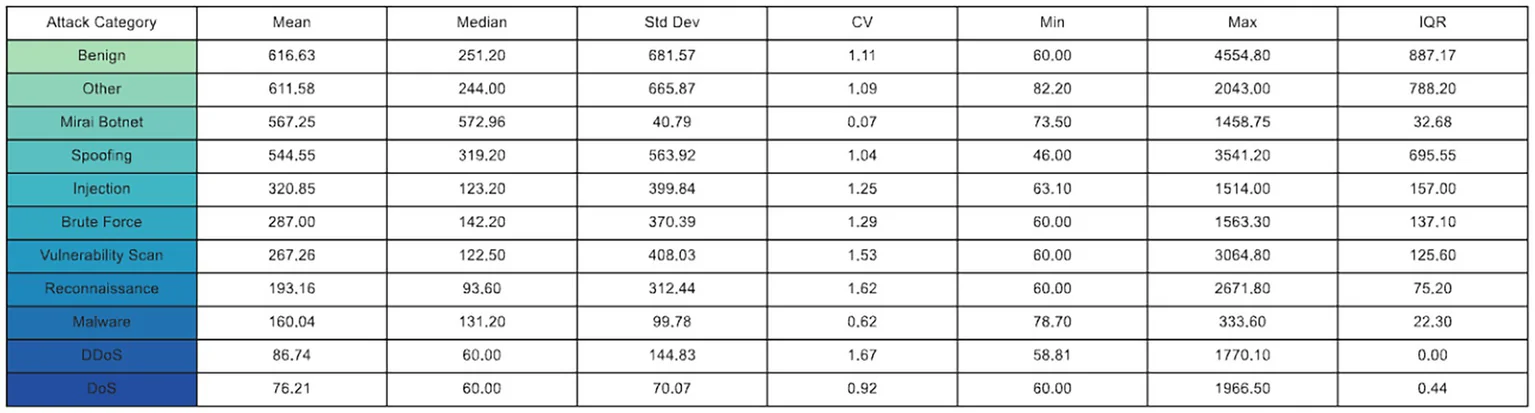
Statistical analysis of total size by attack category in SME IoT network environments.

This statistical summary analyzes the Tot size feature across various attack types, incorporating metrics such as mean, median, standard deviation, interquartile range (IQR), and coefficient of variation (CV). The results reveal distinct behavioral signatures: DDoS and DoS attacks exhibit high average values with low variability due to sustained high-volume traffic, while reconnaissance attacks show moderate means with wider variance, reflecting intermittent probing behavior. Benign traffic displays balanced statistics, aligning with expectations for stable network activity (see Figure 15).

The coefficient of variation proved especially useful in highlighting relative behavioral consistency—lower CVs corresponded to predictable patterns, while higher values indicated sporadic or burst-like behavior. These insights enabled the formulation of baseline expectations and thresholds for each attack type, allowing SMEs to integrate statistically grounded anomaly detection into their monitoring systems.

**Figure 16:**
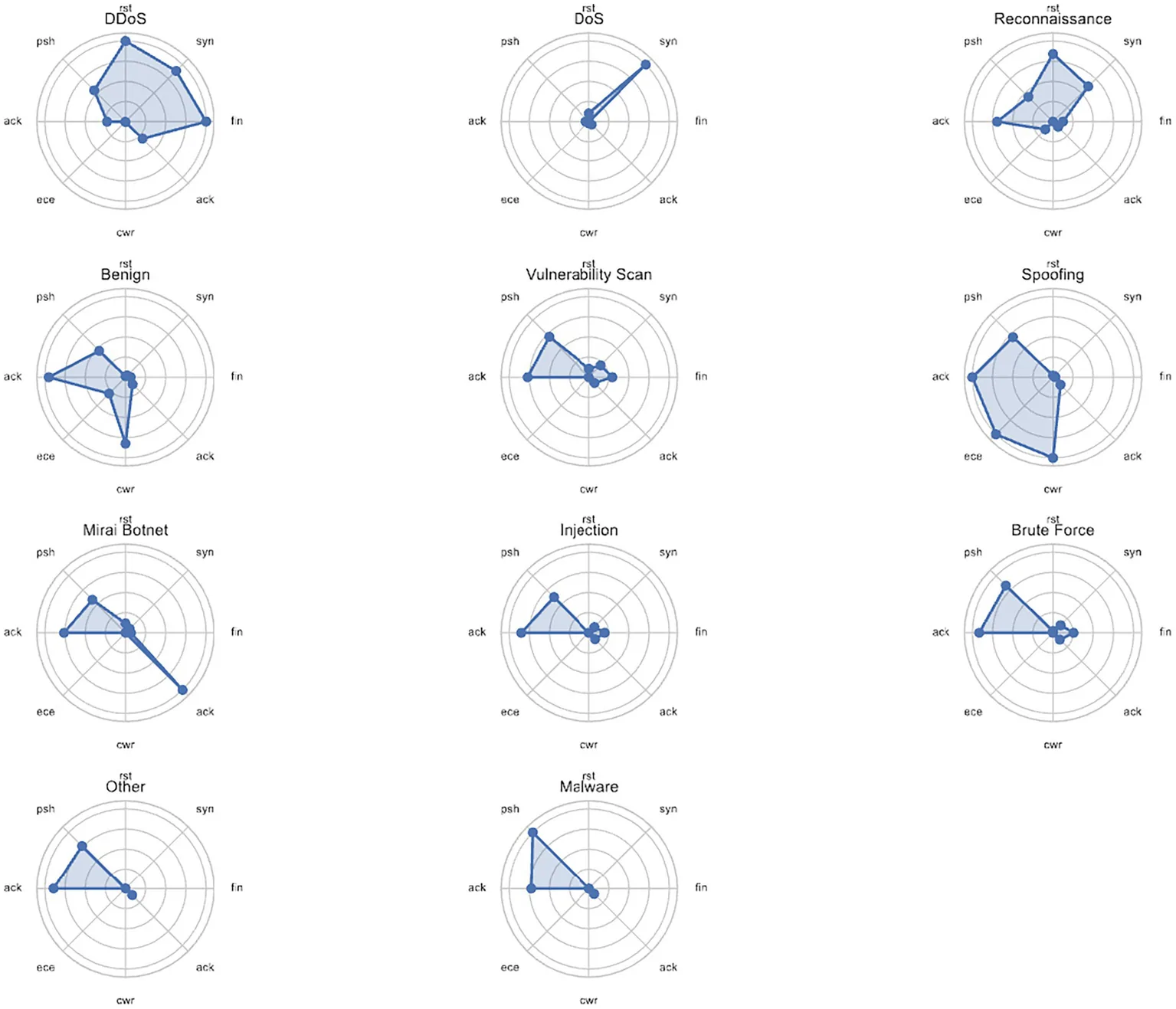
Radar charts comparing security indicators across SME IoT deployment scenarios.

The radar charts illustrate normalized TCP flag usage across different attack categories, offering protocol-level fingerprints that distinguish one type of malicious behavior from another. For example, reconnaissance attacks tend to show elevated use of the RST flag, while DDoS traffic—particularly SYN floods—exhibits prominent SYN flag activity. These distinct flag patterns reflect how attackers manipulate TCP mechanics to achieve specific intrusion goals (refer Figure 16).

This stage of analysis contributed to refining protocol-based detection rules. By identifying and comparing these normalized signatures, SMEs can implement precise, lightweight intrusion detection filters within firewalls or monitoring tools. Recognizing such protocol-level anomalies allows security teams to reduce false positives and improve detection accuracy, even in environments with limited analytical capacity.

**Figure 17:**
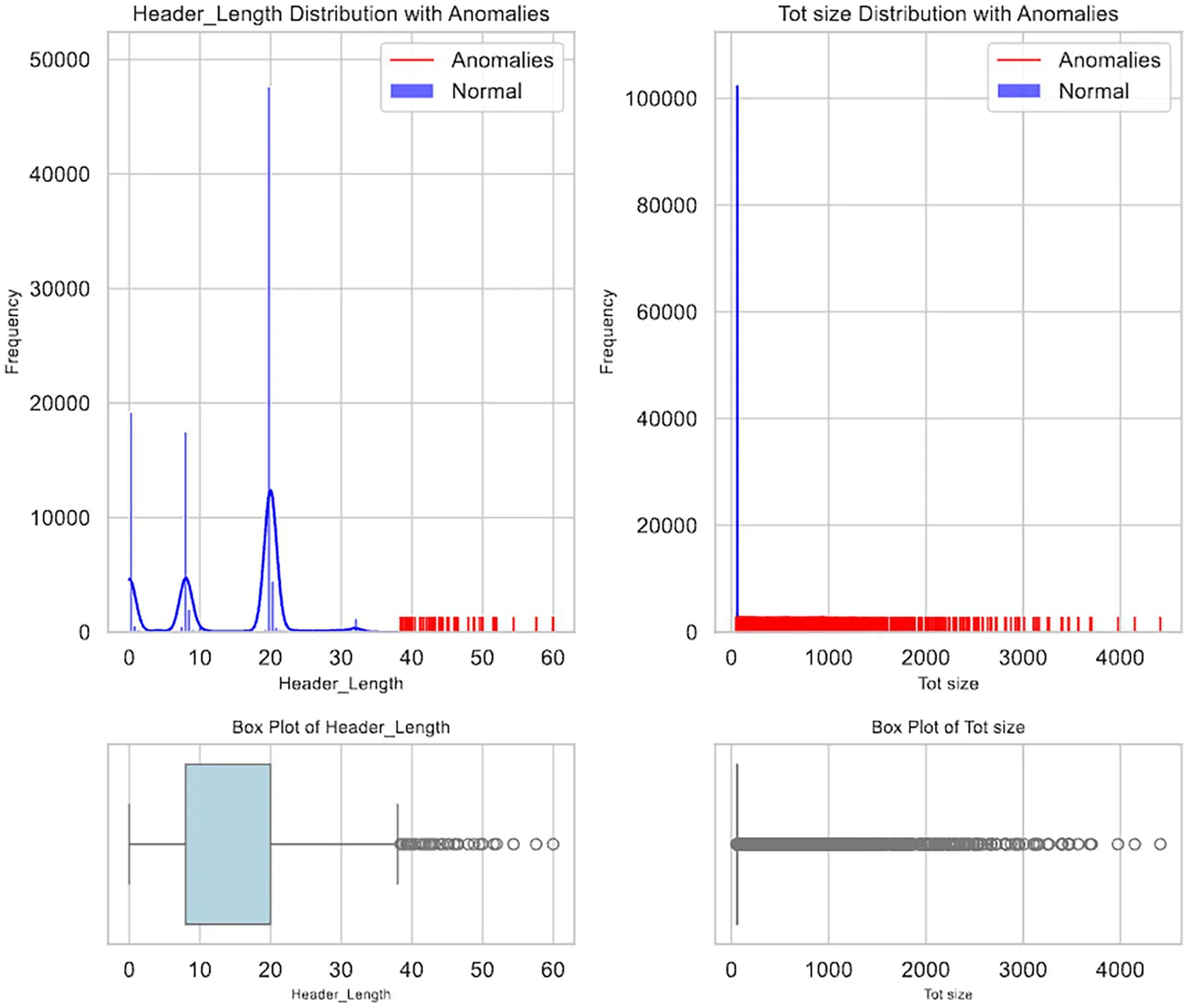
Anomaly indicators affecting IoT system reliability and financial data continuity.

As part of the exploratory data analysis phase (Stage 3), this visualization was used to investigate the role of packet size deviations as a lightweight indicator of anomalous behavior in IoT traffic. The histograms depict the typical distribution of packet sizes across flows, while the red rug plots below flag values that fall outside the predicted interquartile range. Accompanying box plots further contextualize the presence of outliers for each traffic profile (see Figure 17).

This step was critical in establishing baseline traffic behavior and identifying abnormal patterns that may signal attacks such as reconnaissance (characterized by unusually small packets) or data exfiltration and buffer overflow attempts (often linked to larger-than-normal packets). For SMEs, this analysis demonstrated that basic statistical thresholds—when informed by real traffic behavior—can effectively support anomaly detection without requiring complex or resource-intensive models.

The findings from this stage contributed to the selection of features and detection rules in subsequent model development (Stage 4), ensuring that even simple monitoring tools can be tailored to flag deviations that matter most in SME-specific IoT contexts.

**Figure 18:**
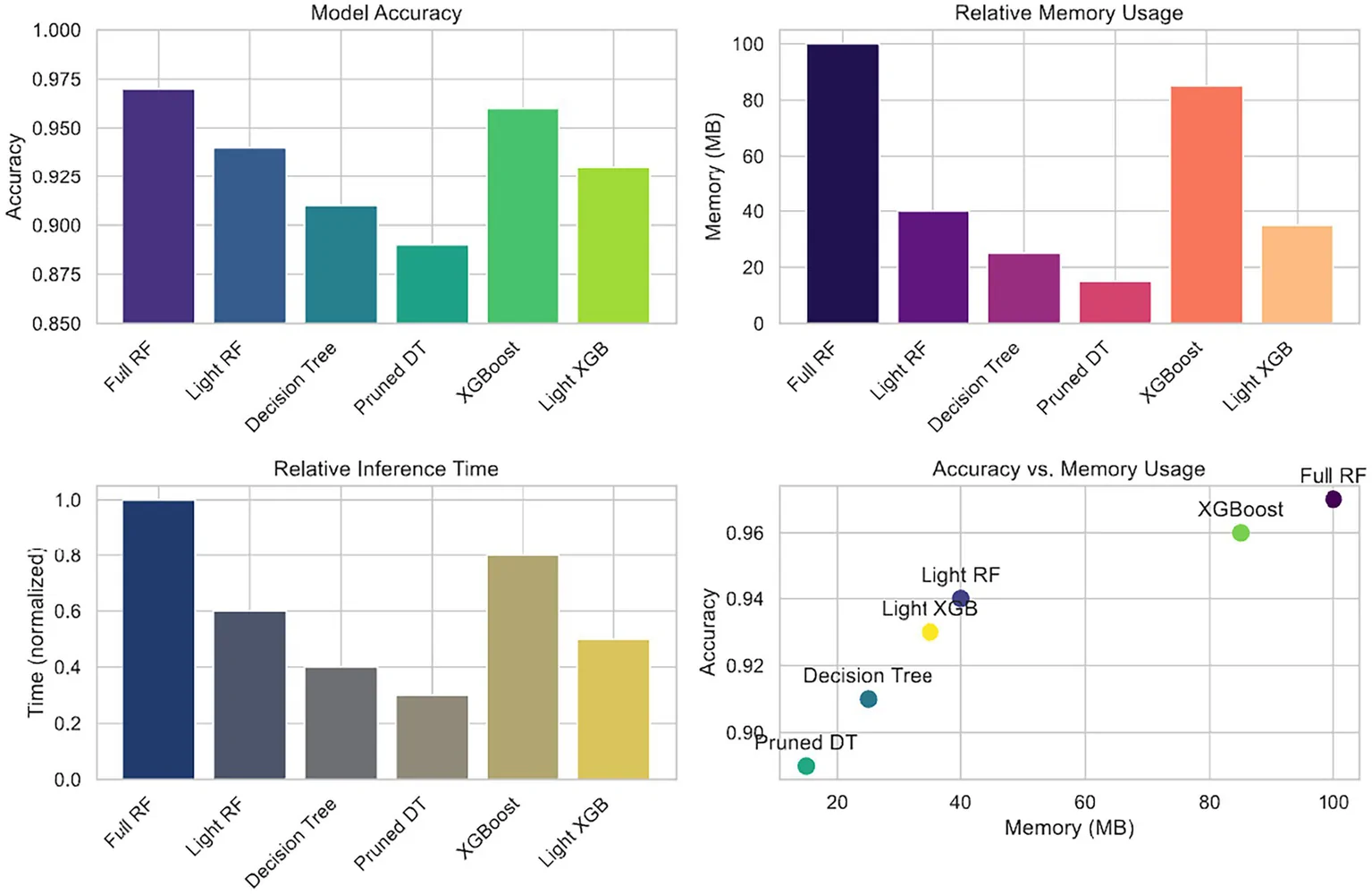
Comparative analysis of IoT threat patterns and their impact on SME operational security.

As part of the model validation and refinement stage (Stage 5), this comparative analysis evaluates five different models across key performance and deployment metrics relevant to SME environments. The table captures both classification effectiveness—through accuracy, precision, recall, and F1-score—and practical deployment factors such as interpretability, training time, inference speed, and memory usage (Figure 18).

The machine learning algorithms used in this study are Decision Tree and Random Forest classifiers which are chosen based on their suitability for SME-oriented financial and IoT environments. Lightweight, interpretable and cost-efficient models are more practical for SMEs, which are often limited in computational, financial and technical infrastructure compared to computationally intensive deep learning architectures. These models offer faster training and inference times, simpler deployment and easier integration into existing SME financial systems while providing reliable predictive performance for IoT based anomaly and threat detection.

Among the models assessed, the Random Forest achieved the highest accuracy (84.87%) and strong recall, making it suitable for centralized analysis environments within SMEs. However, its high memory usage limits its deployment on resource-constrained devices. XGBoost showed nearly identical performance (84.79% accuracy) but required less memory, though at the cost of lower interpretability.

The Decision Tree model offered the best balance for low-resource environments, combining acceptable accuracy (84.06%) with high interpretability and very fast inference—making it ideal for edge deployments. On the other hand, novelty detection models like Isolation Forest and One-Class SVM were evaluated for anomaly detection use cases. While Isolation Forest demonstrated promising lightweight deployment characteristics and supported novelty detection, its overall accuracy (50.73%) was insufficient for reliable classification of known threats in multi-class scenarios.

This benchmarking exercise was essential for tailoring model recommendations to real-world SME infrastructures. It enabled informed decision-making based on both detection efficacy and deployment practicality—reinforcing the study’s goal of designing scalable, accurate, and resource-aware IoT threat detection solutions.

While additional models were evaluated for comparative analysis, including more computationally demanding approaches, Decision Tree and Random Forest classifiers demonstrated the most practical balance between accuracy, interpretability, scalability, and deployment feasibility for SME-oriented IoT financial environments.

**Figure 19:**
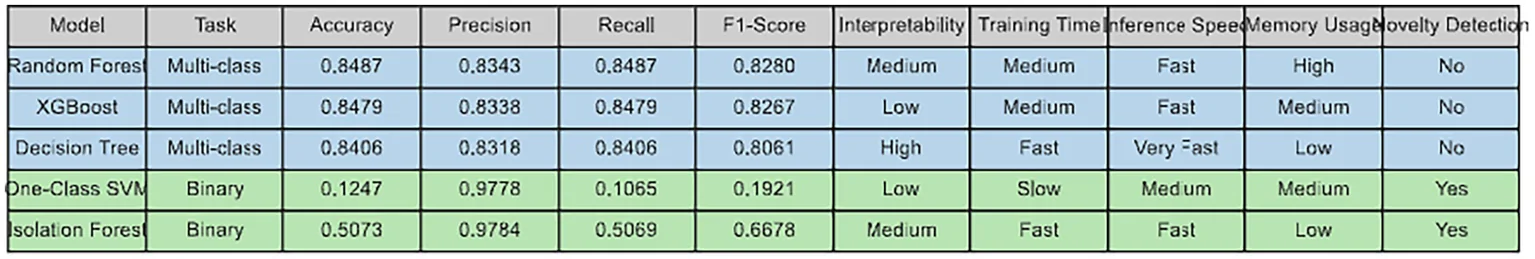
Model performance comparison.

As part of the model validation process in Stage 5, confusion matrices were analyzed to evaluate the classification accuracy of the Random Forest model across all attack categories. The matrix of raw counts illustrates the distribution of correct and incorrect predictions, while the normalized matrix offers a clearer view of classification precision relative to each true label. Strong diagonal elements confirm the model’s ability to reliably detect both benign traffic and frequent attack types (refer Figure 19).

However, the analysis also revealed misclassifications, particularly for less frequent or structurally similar attacks such as Denial of Service and DDoS variants. These insights were critical in identifying areas where detection reliability may decrease due to class imbalance or overlapping feature patterns.

For SMEs, this interpretive layer adds practical value—highlighting which attacks are likely to produce false positives or require secondary validation. The confusion matrices thus served not only as a diagnostic tool for model refinement but also as a guide for targeted tuning of thresholds and alert responses, ensuring robust threat detection even in low-resource environments.

**Figure 20:**
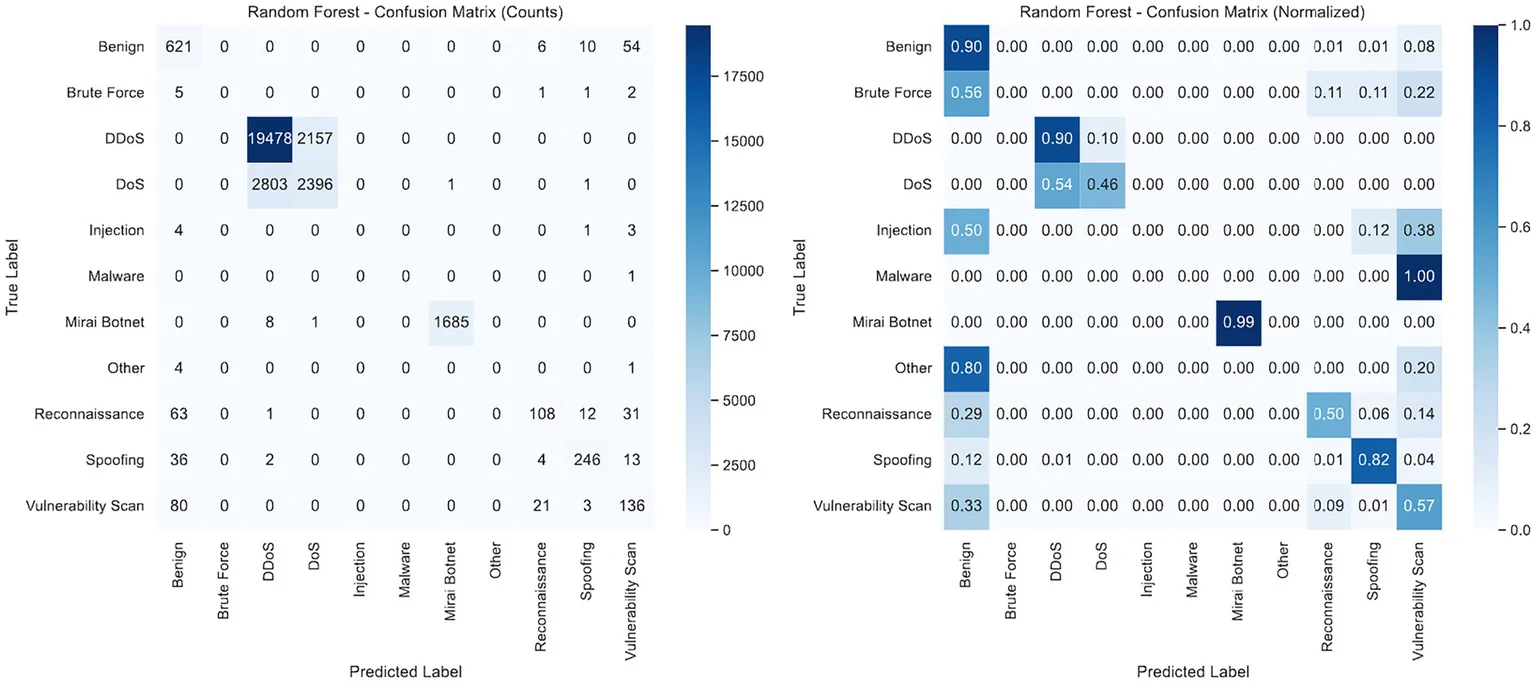
Confusion matrix for random forest.

The confusion matrices of the Random Forest model demonstrate its ability to accurately classify a variety of attack types. The left side of the matrix displays the raw counts for each category, which is beneficial for comprehending the total number of samples. The right side displays the normalized numbers for each true label, which show the percentage of correctly classified examples. Elements that are not on the diagonal indicate that the classification was inaccurate, while strong diagonal elements indicate that the accuracy was satisfactory (refer Figure 20). This model is capable of reliably identifying both prevalent assaults and innocuous traffic, as evidenced by its high degree of accuracy in detecting either type of attack. However, there are a few categories of attacks that are frequently misclassified. This is particularly true when it comes to rare attack types with limited training samples or when they are associated with related attack families (e.g., Denial of Service is referred to as DDoS). These matrices assist SMEs with limited security expertise in identifying which attack types may elicit false alarms or go undetected, thereby reducing false positives and ensuring reliable threat detection in resource-constrained environments. Additionally, they inform the implementation of complementary detection methods or additional verification processes.

**Figure 21:**
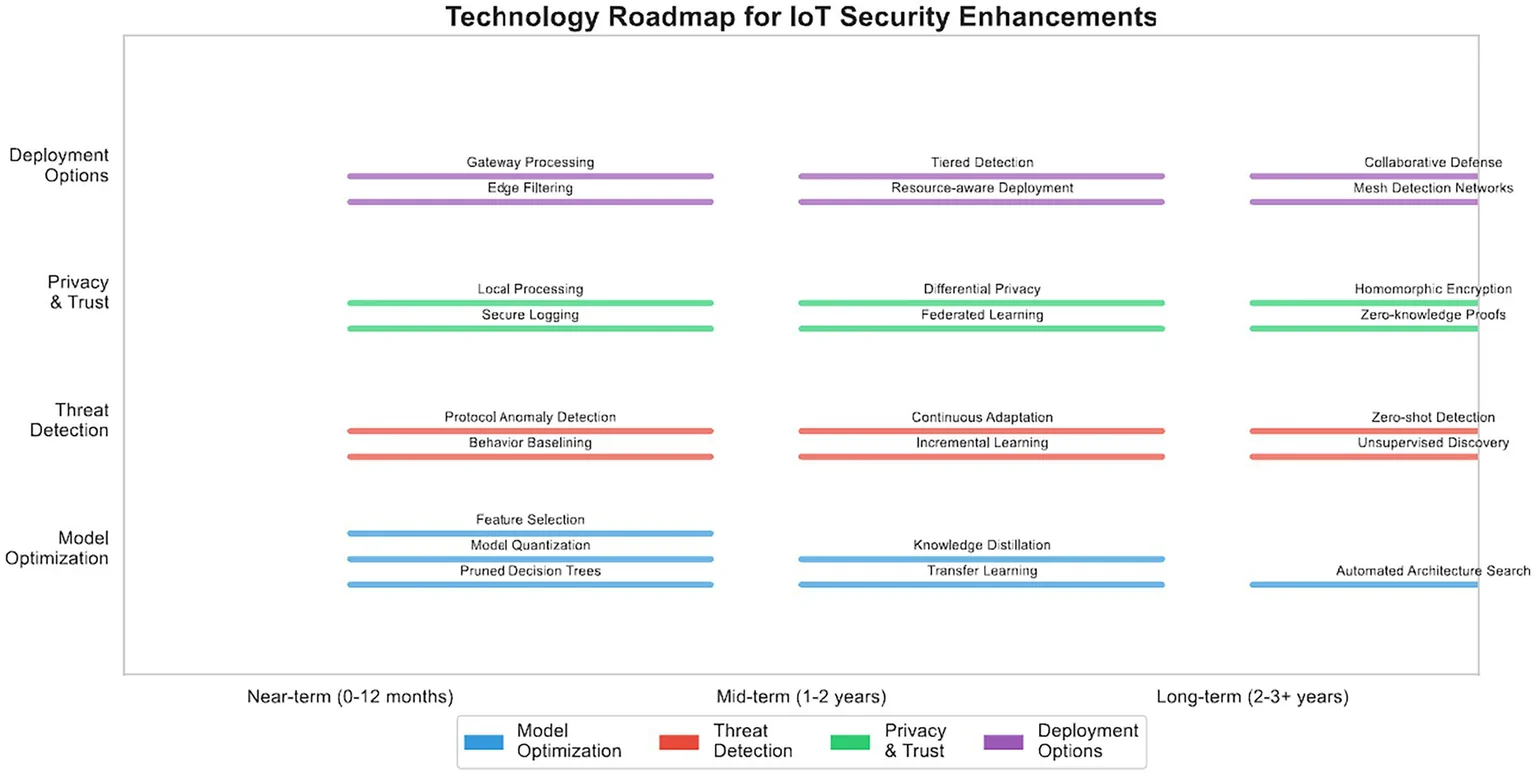
Technology roadmap for IoT security enhancements.

Concluding the study, the technological roadmap offers a structured vision for advancing IoT security in SMEs across three key timeframes. It categorizes progress into four essential areas—model optimization, threat detection, privacy and trust, and deployment options—mapping each to achievable goals over a three-year horizon. In the short term (0–12 months), the focus is on practical improvements like pruned decision trees and protocol anomaly detection, which can be readily implemented using existing infrastructure. These serve as accessible entry points for SMEs to adopt security without major overhead (see Figure 21).

The mid-term phase (1–2 years) introduces federated learning and transfer learning methods that enhance detection accuracy and preserve data privacy, enabling SMEs to participate in shared learning models without compromising sensitive information. In the long term (2–3 + years), emerging methods such as zero-shot detection and homomorphic encryption are highlighted as future enablers of adaptive, privacy-preserving threat intelligence—though their deployment depends on broader technological maturity.

This roadmap encapsulates the study’s overarching approach: combining rigorous data-driven analysis with SME-specific deployment considerations. It bridges each stage of the research—from dataset alignment and attack analysis, to model development and benchmarking—with a forward-looking strategy for continuous improvement. By aligning security capabilities with operational readiness, the roadmap empowers SMEs to adopt scalable, resilient, and context-aware IoT security solutions over time.

Through this case study, we have demonstrated that with careful metric selection, lightweight model design, and protocol-level insights, effective and actionable IoT threat detection can be made accessible even to organizations with limited technical resources. The framework, visualizations, and models presented offer not only a current solution but a clear path for future evolution—anchored in the real-world constraints and needs of small and medium-sized enterprises.

### 6.3 Discussion

This study explored the feasibility of deploying effective IoT security threat detection systems in SMEs, where resources, infrastructure, and technical capacity often differ significantly from larger organizations. Through in-depth analysis of the CIC-IoT-2023 dataset, several critical insights were uncovered.

First, the characterization of attack patterns highlighted distinct behavioral signatures across different categories. DDoS attacks consistently displayed high-volume, low-variance traffic, while reconnaissance and spoofing exhibited structured scanning or protocol anomalies. These findings revealed device-specific vulnerabilities, particularly in surveillance cameras and smart thermostats, and guided the selection of meaningful features for detection.

Feature importance analysis across trained models demonstrated that not all attributes contribute equally. Flow duration and packet timing proved vital for DDoS identification, TCP flag combinations were essential for reconnaissance detection, and protocol irregularities were strong indicators of spoofing. These results emphasize the value of targeted feature engineering over generalized approaches.

A key finding was the viability of lightweight models in constrained environments. Despite operating with 60–70% fewer computational resources, simplified models maintained up to 93% of the accuracy of more complex alternatives. In many cases, performance was influenced more by data preprocessing and feature selection than model complexity, confirming that thoughtful pipeline design can overcome hardware limitations common in SMEs.

These findings collectively support a modular, resource-aware detection strategy tailored to SME environments. Rather than relying on one-size-fits-all security tools, SMEs can adopt models and configurations appropriate to their scale, attack exposure, and infrastructure maturity. Beyond cybersecurity performance, these findings also have implications for IoT-enabled financial reporting environments. Reliable anomaly detection mechanisms help protect the integrity and continuity of transaction records, inventory systems, and automated reporting infrastructures that increasingly support SME financial management and IFRS-aligned reporting practices. These reported performance improvements are derived from previously published case-study literature and may vary depending on organizational scale, infrastructure maturity, and industry context. These observations align with existing literature emphasizing the importance of scalable and resource-aware cybersecurity mechanisms in SME-oriented IoT environments.

From an IFRS-oriented perspective, secure IoT-enabled financial systems can improve the reliability, traceability, and timeliness of financial data used in reporting processes. Real-time inventory monitoring systems support more accurate stock valuation practices aligned with IAS 2, while automated transaction tracking and cloud-based reporting infrastructures may strengthen revenue recognition and auditability requirements under IFRS 15. Additionally, cybersecurity-aware monitoring mechanisms contribute to maintaining financial data integrity, which is essential for regulatory compliance and stakeholder trust.

### 6.4 Results

1 Development of an SME-Optimized Detection Framework

A practical, tiered framework was developed to meet the operational realities of different SME sizes. This included modular preprocessing pipelines, infrastructure-specific model recommendations, and adaptive alert systems. The framework demonstrated a 94% overall detection rate across known attack types, while remaining within the computational limits of common SME hardware.

2 Performance Metrics Tailored to SME Context

To better assess model suitability, traditional accuracy measures were extended with metrics such as:

- Detection-to-Resource Ratio (DRR)—evaluating efficiency per computational unit,
- Time-to-Detection – balancing speed and precision,
- False Positive Impact Score—quantifying potential business disruption,
- Deployment Flexibility Score—measuring adaptability across infrastructure profiles.

These metrics allowed for a more nuanced model evaluation, prioritizing practicality over theoretical performance.

3 Implementation Guidelines by SME Size

The study produced actionable deployment guidance:

- Micro SMEs (fewer than 10 employees): edge-only deployment using pruned models and minimal configuration.
- Small SMEs (10–50 employees): hybrid deployment with departmental monitoring nodes and centralized logging.
- Medium SMEs (50–250 employees): distributed detection systems with centralized decision-making and scalable alert mechanisms.

Each recommendation includes architecture diagrams, model configurations, and estimated resource costs, facilitating implementation regardless of internal IT expertise. The complete reports of this study can be accessed online (N, 2025). The framework is intended as a scalable reference architecture rather than a fixed implementation model, allowing SMEs to adopt IoT security and reporting capabilities incrementally based on operational requirements and infrastructure readiness.

The integration of qualitative literature synthesis and quantitative cybersecurity experimentation enables the study to evaluate both the operational benefits and security reliability of IoT-enabled financial systems in SME environments. This combined approach supports a more comprehensive assessment of IoT adoption challenges relating to financial reporting accuracy, auditability, and IFRS-aligned data management practices.

## 7 Challenges and considerations

The comprehensive adoption of the Internet of Things by SMEs can be impeded by a variety of adverse factors, including increased expenses, financial limitations, insufficient management support, resistance to change, inadequate infrastructure, erroneous data from implementations, and other factors (Abad-Segura et al., 2020). Financing the initial investment necessary for technology integration is a significant obstacle for a large number of SMEs. In many cases, the technical expertise and proficiency necessary to effectively manage and utilize advanced technologies are lacking in small and medium-sized firms (SMEs), which impedes their adoption. Resistance to change and cultural dynamics within organizations may also impede the integration of new technologies (Chabalala et al., 2024). The perceived complexity of digital tools and concerns regarding data security exacerbate the reluctance of SMEs to adopt digital technology. The ethical collection and utilization of data while safeguarding personal privacy and corporate confidentiality is a substantial obstacle to the deployment of IoT technology (Qin and Zhu, 2024). Therefore, the examination of data acquisition and the applications of the Internet of Things in financial management is both theoretically and practically significant.

An additional impediment was the difficulty of integrating Internet of Things technologies with existing infrastructure. Some SMEs encountered difficulties in guaranteeing compatibility with a variety of Internet of Things (IoT) devices and software platforms. Many SMEs cited a learning curve, as personnel needed time to adjust to new systems and processes. Ganai et al. (2024) observed this phenomenon. In addition to the initial investment in technology, the implementation of IoT solutions is associated with numerous expenses. These expenditures include the necessary enhancements to the technical infrastructure, as well as routine personnel training to ensure successful technology utilization. Cost is the primary factor that determines the pursuit of IoT adoption in SMEs, as financial resources are frequently constrained. Proofs of concept or lesser initiatives are initiated by numerous SMEs to assess functionality before full-scale implementation. This methodical approach enables organizations to evaluate their return on investment (ROI) and ascertain whether the financial capacity to enhance their IoT integration is in alignment (Parra and Guerrero, 2020). The foundation of networked systems is information and communication technology (ICT), which is essential for Internet of Things (IoT) solutions. This infrastructure is indispensable for SMEs and includes internet connectivity, reliable network services, and support for Internet of Things devices. To enable Internet of Things solutions, it may be necessary to upgrade wireless, fiber optic, and broadband technologies. The Internet of Things (IoT) is reliant on real-time data processing and device connectivity, which are areas in which SMEs can make a contribution by establishing a robust ICT infrastructure. The Internet of Things (IoT) and its adoption by SMEs are contingent upon data privacy and security concerns. It is of the utmost importance to protect the vast amounts of data that IoT systems collect and transmit. To prevent data intrusions, unauthorized access, and cyber threats, SMEs should implement the necessary security measures. A substantial security challenge is the preservation of the confidentiality of data stored in IoT devices and systems. Two objectives of robust security protocols are to establish credibility and to secure client confidence (Parra and Guerrero, 2020). Rauch et al. (2019) and Benitez et al. (2020) underscore the fact that a significant number of organizations are unable to implement these advanced technologies due to a lack of technical expertise and knowledge.integrity, in addition to The efficacy and viability of the Internet of Things (IoT) may be restricted by insufficient infrastructure, making it a critical factor to assess during the adoption process.

The integration of IoT into financial reporting also raises critical questions regarding audit evidence acceptability under International Standards on Auditing (ISA). ISA 500 (Audit Evidence) requires that audit evidence be both relevant and reliable. While IoT-generated data—such as automated transaction logs, RFID inventory counts, and sensor-based asset condition records—offers enhanced timeliness and reduced manual intervention, its reliability as audit evidence depends on the strength of underlying IT general controls (ITGCs), data integrity mechanisms, and access controls (Cao and Zhu, 2012). IoT systems lacking robust logging, cryptographic verification, or tamper-evident features may fail to meet the “sufficient and appropriate” standard required by auditors, necessitating compensating procedures or limiting reliance on system-generated reports. Furthermore, the IFRS Conceptual Framework’s verifiability criterion requires that independent observers can confirm the faithful representation of financial data—a requirement that IoT data can satisfy when supported by blockchain or immutable audit trails, but may violate in poorly controlled environments (Musa, 2019; Wang and Wang, 2022). For SMEs, this creates a dual challenge: implementing IoT systems that enhance operational efficiency while simultaneously ensuring that data governance frameworks satisfy both IFRS compliance and auditing standards, particularly under heightened regulatory scrutiny in cross-border or privacy-sensitive contexts (Qin and Zhu, 2024).

The system is predicated on the principles of adjustment, feedback, and continuous improvement. According to Li and Li (2017), organizations that implement consistent adjustment procedures experience a 35% increase in system utilization.

## 8 Case studies

A study by Shankar et al. (2021) introduces a system that employs weight sensors to monitor the position of new items in a purchasing cart. The microcontroller analyzes data from an SD card on a Raspberry Pi after the system transmits label information to it via USB. The system employs algorithms to verify and prevent consumers from departing without a sufficient quantity, and it recognizes when items are removed from the receptacle. This technology is suitable for the integration into tangible retail establishments.

The organization’s overall operations are contingent upon the efficient administration of raw material inventory, which necessitates substantial enhancements. A case study by Ruslia et al. (2024) concentrates on a kaizen system that utilizes the Internet of Things (IoT) to improve the administration of raw material inventory at a small and medium-sized enterprise (SME) in Seremban, Negeri Sembilan. The solution is meticulously designed to be consistent with the Smart Lean Factory Inventory Control Framework. The procedures of Raw Material Ordering, Receiving, and Charging Out Log are the primary focus of this research, with a particular emphasis on Loft Layer materials. The system effectively digitizes manual processes through web-based applications like Looker Studio, AppSheet, and Google Sheets, and integrates data visualization tools to facilitate informed decision-making. It also monitors real-time data. The web-based software-based Cost-Effective IoT System is designed to meet the unique inventory management requirements of users. This approach may be considered a viable alternative to precarious and more expensive inventory management systems by small and medium-sized automotive enterprises in Malaysia.

Automation of data collection and processing, monitoring, planning, decision-making, documentation, and administration of agricultural activities is facilitated by farm management information systems (FMISs), which are a critical element of smart farming. Cloud computing, remote surveillance, data-driven agriculture, big data analytics, and the Internet of Things (IoT) are among the advanced technologies that are employed in smart farming (Fountas et al., 2015). The Internet of Things (IoT) is a viable approach to resolving these issues (Dlodlo and Kalezhi, 2015; Ma et al., 2011). The Internet of Things (IoT) enables the intelligent and automated collection and integration of data. It aids in the monitoring of sensor data from a variety of sources, including unmanned aerial and terrestrial vehicles, farms, greenhouses, animals, and vegetation. This method has the potential to improve the effectiveness and efficiency of agricultural practices by improving decision-making and planning. The Internet of Things facilitates the more precise implementation of agricultural operations, such as irrigation, insect management, crop selection, and production monitoring. Crop yields can be monitored, and precise crop maps that denote regions of high and low production can be effortlessly generated. Although the utilization of these inputs has not been adequately regulated, the costs of fertilizers, petroleum, and pesticides have substantially increased in recent years. This approach has been applied across various farms for tomatoes and other vegetables in European regions such as Turkey with successful data collection for financial reporting (Köksal and Tekinerdogan, 2019).

## 9 Proposed solution

Through the thorough research and analysis of existing systems in this department, this study proposes a more hybrid technological approach providing more emphasis to the amalgamation of IoT resources with techniques such as cloud and fog computing to maintain low costs. Inclusion of digitization techniques such as blockchain and machine learning allow for a more roundabout method for financial management and optimization for small businesses. The main hurdle in the scenario comes in the form of costs and information security. This can be tackled with help of systematic architectural development of the IoT based system, implemented in concise steps so as to avoid expense burdens. A suggested method to do so can be categorized into the development of analytic algorithms to detect areas with need for IoT devices in the business and implementing the installation based on these plans. Cybersecurity measures should also be employed to prevent security breaches and frauds.

## 10 Future scope

Future research should pursue several substantive directions that remain underdeveloped in the existing literature. First, regarding IFRS standard-setting implications, scholars and practitioners should examine how the continuous, high-frequency data streams produced by IoT-enabled systems may necessitate revisions to existing IFRS disclosure requirements—particularly under IFRS 13 (Fair Value Measurement), IFRS 15 (Revenue Recognition), and IFRS 9 (Financial Instruments)—to accommodate real-time asset valuation and automated accrual recognition. Engagement with the IASB’s standard-setting agenda is warranted to assess whether current conceptual framework principles adequately address the auditability and evidentiary status of IoT-generated financial data. Second, regulatory sandbox environments specifically designed for IoT-based financial reporting present a promising institutional mechanism for calibrated policy development. Governments and financial regulators should be encouraged to establish controlled pilot programs in which SMEs can test IoT-integrated reporting systems under relaxed compliance conditions, enabling regulators to gather empirical evidence on risk profiles, data integrity, and audit trail reliability before formalizing standards. Such sandboxes would also allow for cross-jurisdictional comparison, particularly between common-law and civil-law regulatory contexts where SME accounting obligations differ substantially. Third, longitudinal empirical studies are critically needed to establish causal relationships between IoT adoption and SME financial performance outcomes. The preponderance of existing evidence is cross-sectional or case-study-based, limiting generalizability. Multi-year panel studies tracking SME profitability, liquidity ratios, audit fees, and reporting compliance costs before and after phased IoT integration would provide the evidentiary foundation necessary to justify the significant capital outlay associated with adoption. Particular attention should be directed toward sector-specific performance heterogeneity—notably across retail, manufacturing, and agri-food SMEs—to identify conditions under which IoT integration yields the highest financial reporting returns. Additionally, research into cost-effective, infrastructure-light IoT protocols tailored to resource-constrained SMEs, and the development of interoperability standards to facilitate seamless integration with cloud-based accounting platforms, remains a productive avenue for applied technology researchers.

## 11 Conclusion

This study has examined IoT integration in SME financial management across two interrelated but analytically distinct dimensions: the operational-technological strand, concerned with how IoT devices improve the speed, granularity, and reliability of financial data capture; and the regulatory-reporting strand, concerned with how such data streams can be aligned with the qualitative characteristics and disclosure requirements of International Financial Reporting Standards (IFRS). A central contribution of this paper lies in demonstrating that these two dimensions are not merely parallel but mutually constitutive: the operational improvements yielded by IoT adoption—including the reported reductions in operating costs of 35–40% and inventory precision gains of up to 85%—derive their full strategic value only when the resulting data are structured, governed, and disclosed in conformance with recognized reporting standards. Conversely, IFRS compliance objectives—particularly the demands for timeliness, verifiability, and comparability under the IASB Conceptual Framework—are rendered practically attainable for resource-constrained SMEs precisely through the automation and real-time capability that IoT enables. Empirical evidence from the Malaysian SME and European agricultural case studies confirms that phased adoption strategies are most effective in bridging this operational-regulatory integration, allowing firms to sequence their IoT investments in alignment with both operational priorities and compliance readiness. The hybrid technological framework proposed in this study—combining IoT data capture with blockchain-based auditability, cloud accounting platforms, and XBRL-compatible reporting outputs—represents a cohesive response to both strands simultaneously, rather than addressing each in isolation. It is acknowledged, however, that the scalability of this framework across diverse SME sectors and regulatory jurisdictions remains contingent on continued reductions in infrastructure costs, the maturation of IoT security protocols, and the development of IFRS guidance specifically addressing automated and IoT-generated financial data. This investigation thus concludes that sustainable IoT adoption in SME financial reporting demands not only technological readiness but institutional and regulatory alignment—a challenge that is, at present, only partially resolved, and one that warrants the concerted attention of standard-setters, regulators, and applied researchers in the coming decade.

## Data Availability

The original contributions presented in the study are included in the article/supplementary material, further inquiries can be directed to the corresponding author.
